# Persistent increase of accumbens cocaine ensemble excitability induced by IRK downregulation after withdrawal mediates the incubation of cocaine craving

**DOI:** 10.1038/s41380-022-01884-1

**Published:** 2022-12-08

**Authors:** Guanhong He, Ziqing Huai, Changyou Jiang, Bing Huang, Zhen Tian, Qiumin Le, Guangyuan Fan, Haibo Li, Feifei Wang, Lan Ma, Xing Liu

**Affiliations:** 1 School of Basic Medical Sciences, State Key Laboratory of Medical Neurobiology, MOE Frontiers Center for Brain Science, Institutes of Brain Science, Department of Neurology, Pharmacology Research Center, Huashan Hospital, Fudan University, Shanghai, 200032 China; 2 Research Unit of Addiction Memory, Chinese Academy of Medical Sciences (2021RU009), Shanghai, 200032 China; 3 Present Address: Westlake Laboratory of Life Sciences and Biomedicine, Hangzhou, 310024 China

**Keywords:** Neuroscience, Addiction

## Abstract

The incubation phenomenon, cue-induced drug craving progressively increasing over prolonged withdrawal, accounts for persistent relapse, leading to a dilemma in the treatment of cocaine addiction. The role of neuronal ensembles activated by initial cocaine experience in the incubation phenomenon was unclear. In this study, with cocaine self-administration (SA) models, we found that neuronal ensembles in the nucleus accumbens shell (NAcSh) showed increasing activation induced by cue-induced drug-seeking after 30-day withdrawal. Inhibition or activation of NAcSh cocaine-ensembles suppressed or promoted craving for cocaine, demonstrating a critical role of NAcSh cocaine-ensembles in incubation for cocaine craving. NAcSh cocaine-ensembles showed a specific increase of membrane excitability and a decrease of inward rectifying channels Kir_2.1_ currents after 30-day withdrawal. Overexpression of Kir_2.1_ in NAcSh cocaine-ensembles restored neuronal membrane excitability and suppressed cue-induced drug-seeking after 30-day withdrawal. Expression of dominant-negative Kir_2.1_ in NAcSh cocaine-ensembles enhanced neuronal membrane excitability and accelerated incubation of cocaine craving. Our results provide a cellular mechanism that the downregulation of Kir_2.1_ functions in NAcSh cocaine-ensembles induced by prolonged withdrawal mediates the enhancement of ensemble membrane excitability, leading to incubation of cocaine craving.

## Introduction

A major challenge for the treatment of cocaine addiction is that cocaine craving is often precipitated even after a prolonged period of abstinence by the presentation of drug-associated cues [1, 2]. This “incubation” phenomenon, the progressive increase in cocaine craving occurring after abstinence, is an important contributing factor to the high rates of relapse [3–5]. The discoveries of whether and how the neuroadaptations produced during the drug-free period contribute to the increase of cocaine craving have significant implications for further understanding of the mechanisms underlying drug addiction and relapse in addicts.

After withdrawal, neuroadaptations occur in the nucleus accumbens (NAc), a central region within mesolimbic reward circuits [5–7]. GluA2-lacking calcium-permeable AMPARs (CP-AMPARs) form and accumulate in the NAc after prolonged withdrawal from cocaine self-administration (SA), and cocaine-generated silent synapses mature by recruiting CP-AMPARs resulting in incubation of cocaine craving [8, 9]. The activation of Ca^2+^/calmodulin-dependent protein kinase II signaling, reduces metabotropic glutamate receptor 1 tone, and elevation of BDNF protein in the NAc contribute to incubation of cocaine craving [10–12]. An aberrant increase in protein translation in the NAc is required for incubation of cocaine craving [13]. Withdrawal from cocaine time-dependently enhances DNA methylation in the NAc, which contributes to increased cue-induced cocaine seeking [14]. However, the neuroadaptations driven by cocaine withdrawal are still largely unknown.

The NAc primarily contains D1 and D2 dopamine receptor expression medium spiny neurons (D1-MSNs and D2-MSNs) whose hyperexcitability has been suggested to be presented in cocaine exposure [15, 16]. Membrane excitability, an intrinsic physiological property of neurons, is determined by a set of potassium (K^+^) channels. At hyperpolarized potential, inwardly-rectifying K^+^ (Kir) channels open and comprise the primary conductance that sets the threshold for spike generation and controls signal transmission [17]. Among Kir family, Kir2 subfamilies with high expression levels in the striatum encode classical strong inward rectification [18]. Study shows that footshock stress increases intrinsic excitability of D1-MSNs via reduced Kir activity, and restoring Kir2.1 function in D1-MSNs prevents stress induced negative affective states [19]. Downregulation of NAc MSN excitability by the expression of Kir2.1 enhances locomotor activity induced by cocaine [20]. Recent studies suggest a critical role of neuronal ensembles activated by the presentation of drug cues after voluntary abstinence in incubation expression of nicotine, heroin, and methamphetamine craving [21–23]. It is unknown whether withdrawal from drug can drive excitability changes in neuronal ensembles activated by initial drug exposure and how such changes may influence incubation of drug craving.

In the present study, we assessed activity dynamics of NAcSh cocaine-ensembles labeled by cocaine-SA training during prolonged withdrawal, examined the roles of NAcSh cocaine-ensembles in incubation for cocaine craving, and investigated the molecular changes in cocaine-ensembles occurring during withdrawal that might mediate incubation of cocaine craving.

## Methods

Full methodological descriptions can be found in the Supplementary information.

### Animal

Adult male mice (P40-P90) were used for all experiments. All animal treatments were strictly in accordance with the National Institutes of Health Guide for the Care and Use of Laboratory Animals and were approved by Animal Care and Use Committee of Shanghai Medical College of Fudan University.

### Behavioral experiments

Food SA was conducted first to nose poke for food pellets (14 mg, catalog #F05684, Bio-serv, USA) under a fixed ratio 1 reinforcement schedule. Mice that failed in food-SA training were excluded for surgery. Then cocaine SA was carried out in mouse operant chamber. The mice were trained to self-administer cocaine for ten sessions (1 session/day). Each session lasted for 4 h (0.5 mg/kg/infusion) or 6 h (1.5 mg/kg/infusion). Each active nose poke resulted in a cocaine infusion (0.5 or 1.5 mg/kg/infusion, dissolved in 0.9% saline and delivered at 7.97 ul/s) and a tone-light cue (10 s, 2900 Hz tone with cue light above the nose poke while the house light turned off). Active nose poke triggers a timeout period of 40 s. Poking in the inactive hole had no consequences. Mice were randomly assigned to experimental and control groups and the investigator was blind to group allocation.

We assessed incubation of cue-induced cocaine craving in drug-seeking tests conducted in the same mouse after the various times of withdrawal from cocaine SA. During withdrawal, mice were kept in their home cage. The drug-seeking test was performed for 30 min or 60 min, during which, active nose pokes resulted in contingent delivery of the tone-light cue, identical to the cocaine training procedure but without cocaine infusion. Pump noise was maintained.

### Ex vivo electrophysiology recording

Coronal slices (300 μm) containing the NAcSh were prepared as previous [24]. K^+^-based intracellular solution was used for action protential (AP) recording. A current-step protocol (from −40 to +200 pA, with a 20-pA increment) lasting 1 s was run and repeated. Resting membrane potential (RMP) was recorded without current injection. Rheobase was measured by injecting a variable positive current step (2 pA increment from the beginning of 0 pA) lasting 800 ms until the cell discharged a single AP. Membrane input resistance was measured by injecting a negative 100 pA current step lasting 1 s while holding the membrane potential to −80 mV. Inward rectifying K current (I/V curve) was recorded using a 10-mV voltage step from −150 mV to −40 mV while holding the membrane potential to −80 mV. Kir_2.1_ blocker ML133 HCL (50 μM) was bath applied. Recordings with Rs >30 MΩ were excluded from statistical analysis.

### Fiber photometry

An optical fiber was implanted unilaterally into the NAcSh of mice and the fluorescence signals were recorded using a Fiber Photometry system equipped with 470- and 410-nm excitation laser (Inper Tech). Each animal was tested 1 h for 1 trial after 1-day and 30-day withdrawal in a MED-PC operant chamber, respectively, and signals were recorded for the entire duration. The mice with off-target fiber tip were excluded from the analysis.

### Ribosome-associated transcripts

The purification procedure was modified and performed as previously reported [25]. NAcSh cocaine- or saline-ensembles were labeled in cocaine-SA or saline-SA training. Mice were decapitated 1, 30 or 90 days after cocaine-SA or saline-SA training. The brain sections containing the NAcSh were quickly manually dissected and immediately homogenized. Homogenates were centrifuged and the supernatant lysate (output) was incubated with 3 μg Rabbit anti-HA (H6908, Sigma-Aldrich) for 4 h and 100 μl Dynabeads Protein G overnight at 4 °C with end-over-end rotation sequentially. Beads were collected and purified mRNA was eluted from the Dynabeads. Then mRNA was enriched and sequenced.

### Statistical analysis

Our sample sizes were based on our previous research [24, 26]. Single-variable comparisons between groups were analyzed with two-tailed Student’s *t* test or one-way ANOVA. Two-variable comparisons between multiple groups were analyzed using two-way ANOVA, followed by Bonferroni’s post hoc test. Data are presented as mean ± SEM.

## Results

### Prolonged withdrawal predisposes cocaine-ensembles to a state susceptible to activation

We trained mice to self-administer cocaine (0.5 or 1.5 mg/kg/infusion) at a fixed-ratio-1 for 10 daily sessions, during which an active nose poke led to a cocaine infusion paired with a tone-light cue [27]. The drug-seeking tests were performed during withdrawal to access cue-induced cocaine-seeking behavior. Consistent with the reports of incubation of cocaine craving in rats [12] and mouse [27] models, we observed a progressive increase of cue-induced active nose pokes, not the inactive nose pokes, overtime after the cessation of 10-day cocaine-SA training at 1.5 mg/kg/infusion, which reached the peak after 30-day withdrawal (Supplementary Fig. S1a–e), showing incubation of cocaine craving.

**Fig. 1:**
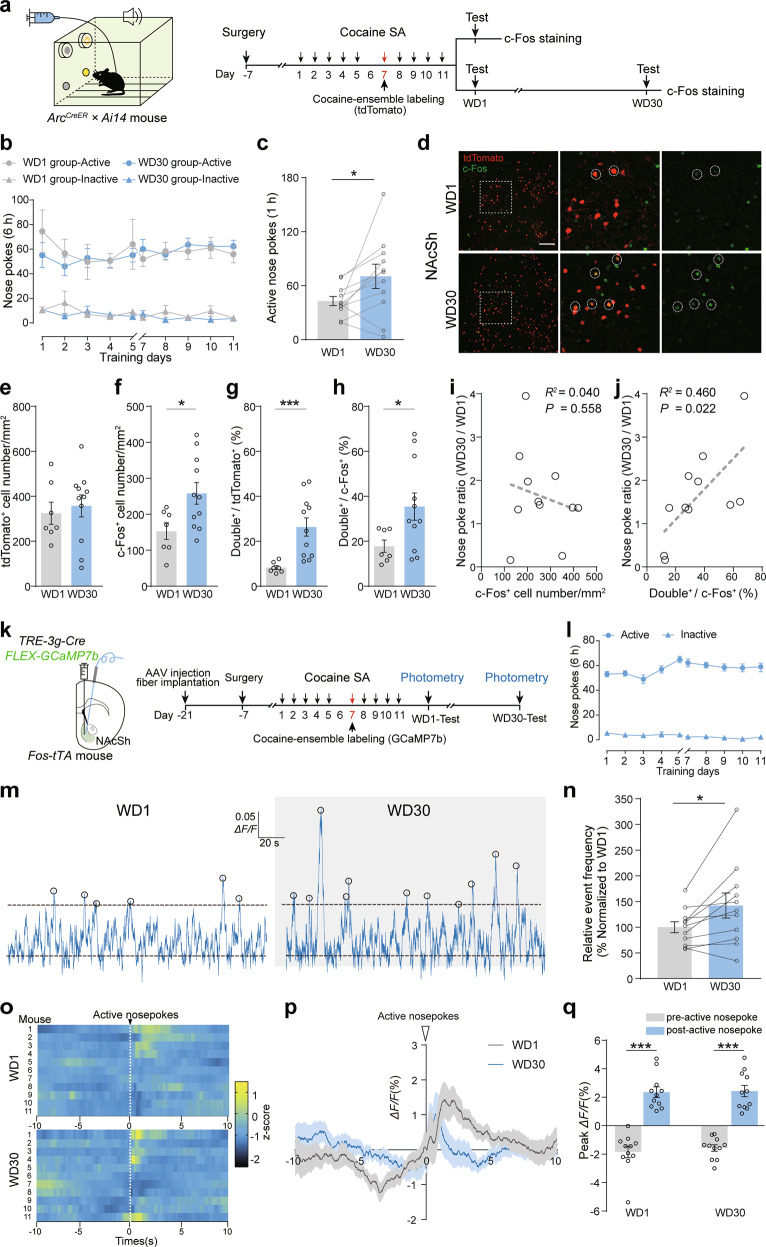
Cue-induced cocaine-seeking behavior and the activity of NAcSh cocaine-ensembles are increased after prolonged withdrawal. **a**–**j** Cocaine-SA training was performed on days 1–5 and 7–11 (1.5 mg/kg/infusion, 6 h/day) in *ArcTRAP;AI14* mice. Tamoxifen (TAM, 125 mg/kg, i.p.) was injected on day 6 to allow *Arc*-driven tdTomato expression (ensemble labeling). The mice were sacrificed 60 min after cue-induced drug-seeking test for c-Fos immunostaining after 1-day and 30-day withdrawal, respectively. **a** Experimental scheme. **b** Plots of active and inactive nose pokes during cocaine-SA training. [*n* = 11, Active nosepokes, *F*_group×session_ (9, 144) = 0.905, *p* = 0.523, Inactive nosepokes, *F*_group×session_ (9, 144) = 1.680, *p* = 0.099, RM two-way ANOVA]. **c** Active nose pokes during drug-seeking test (1 h) after 1-day and 30-day withdrawal. [*n* = 11, *t*_(10)_ = 2.351, *p* = 0.041, Two-tailed paired *t*-test]. **d** Representative images of tdTomato and c-Fos expression in the NAcSh of *ArcTRAP;AI14* mouse. Red: tdTomato; Green: c-Fos. Scale bar: 100 μm. Bar graph of tdTomato^+^ cell number/mm^2^ (**e**), c-Fos^+^ cell number/mm^2^ (**f**), c-Fos^+^ tdTomato^+^/tdTomato^+^ (%) (**g**), and c-Fos^+^ tdTomato^+^/c-Fos^+^ ratio (%) (**h**) in the NAcSh. [WD1: *n* = 7, WD30: *n* = 11; tdTomato^+^ cell number/mm^2^: *t*_(16)_ = 0.448, *p* = 0.660, c-Fos^+^ cell number/mm^2^: *t*_(16)_ = 2.471, *p* = 0.025, Two-tailed Student’s *t* test; c-Fos^+^ tdTomato^+^/tdTomato^+^ (%): *U* = 2, *p* < 0.001, Mann–Whitney *U* test; c-Fos^+^ tdTomato^+/^c-Fos^+^ (%^)^: *U* = 13, *p* = 0.020, Mann–Whitney *U* test]. Correlation of c-Fos^+^ cell numbers/mm^2^ (**i**) or c-Fos^+^ tdTomato^+/^c-Fos^+^ ratio (%) (**j**) with the normalized active nose poke ratio (WD30/WD1) in WD30 group [*n* = 11, **i**: *R*^2^ = 0.040, *p* = 0.558; **j**: *R*^2^ = 0.460, *p* = 0.022]. **k**–**q** *AAV-TRE-3g-Cre and AAV-FLEX-GCaMP7b* were injected in the NAcSh of *Fos-tTA* mice fed on diet containing Dox (40 mg/kg) and subjected to 1.5 mg/kg/injection cocaine-SA training, and the optic fiber was unilaterally implanted for photometry. Regular diet without Dox was provided on days 6–7 to allow *c-fos*-driven expression of GCaMP. Photometry recording was performed during drug-seeking tests after 1-day and 30-day withdrawal. **k** Experimental scheme. **l** Plots of active and inactive nose pokes during cocaine-SA training. **m** Sample GCaMP7b photometry trace of cocaine-ensembles during drug-seeking tests. Circles marked above trace indicate threshold-detected events. **n** Relative event frequency (% Normalized to WD1) of cocaine-ensembles during drug-seeking tests [*n* = 11, *t*_(10)_ = 2.647, *p* = 0.025, Two-tailed paired *t*-test]. **o** Heatmap of GCaMP fluorescence in the NAcSh cocaine-ensembles in response to active nosepokes after 1-day and 30-day withdrawal. **p** Average Δ*F*/*F* (%) of GCaMP fluorescence in response to active nosepokes after 1-day and 30-day withdrawal. **q** Peak Δ*F*/*F* (%) 10 s pre- and post-active nosepokes after 1-day and 30-day withdrawal. [*n* = 11, *F*_withdrawal×session_ (1, 20) = 0.047, *p* = 0.831, RM two-way ANOVA]. *p < 0.05 and ***p < 0.001 vs. indicated group.

To examine the role of the neuronal ensembles activated by cocaine-SA training in cue-induced drug-seeking behavior after the different duration of withdrawal, we utilized activity-dependent, immediate early gene-driven cell-tagging techniques. *ArcTRAP;AI14* mice underwent 10 sessions (1 session/day, days 1–5, and days 7–11) of cocaine-SA training, and on day 6 the mice were injected with Tamoxifen (TAM, 125 mg/kg, i.p.) to label cocaine-SA activated cocaine-ensembles with tdTomato (Fig. 1a, b). Open field test showed that TAM injection did not significantly change locomotor activity 1 day later (Supplementary Fig. S2e). The basal neuronal activity of cocaine-SA ensembles in the NAcSh was kept at low levels and exhibited no difference between 1-day and 30-day withdrawal (Supplementary Fig. S2a–d). After conditioning stimuli-induced drug-seeking test, we found that the c-Fos^+^ cell counts and the percentage of c-Fos^+^ cocaine-ensembles (c-Fos^+^ tdTomato^+^ cells) in the NAc of 30-day withdrawal mice (WD30) were higher than those of 1-day withdrawal mice (WD1) (Fig. 1c–h and Supplementary Fig. S2f–o). NAcSh ensemble activation was enhanced by drug-seeking test after 30-day withdrawal and began to decline after that (Supplementary Fig. S3). Moreover, the extent of cocaine-seeking (nose poke ratio of WD30/WD1) was positively correlated with the ratio of tdTomato^+^ c-Fos^+^/c-Fos^+^, but not c-Fos^+^ cell counts, in the NAcSh of the individual mouse (Fig. 1i, j).

Dopamine receptor D1 and D2 positive medium spiny neurons (D1-MSNs and D2-MSNs) constitute 90% of the NAc neurons [28]. We, therefore, evaluated the proportion of D1 and D2 positive cocaine-ensembles in D1-tdTomato and D2-eGFP transgenic mice. As shown in Supplementary Fig. S4, 75.61% of NAcSh ensembles were D1-MSNs (c-Fos-eGFP^+^ tdTomato^+^) and 15.89% were D2-MSNs (c-Fos-mCherry^+^ eGFP^+^) (Supplementary Fig. S4a–c). This result was confirmed by multiplexed single-molecule RNA fluorescence in situ hybridization (smFISH) of *Drd1* and *Drd2* in *ArcTRAP;AI14* mice (Supplementary Fig. S4d–f). The above data suggest that cocaine exposure and prolonged withdrawal predispose cocaine-ensembles to a state that is hyperactive to drug-conditioned stimuli, and this increased susceptibility to activation is associated with increased craving for cocaine after prolonged withdrawal.

To examine the activity of cocaine-ensembles during drug-seeking test, we expressed GCaMP in NAcSh cocaine-ensembles and recorded the Ca^2+^ events synchronizing with the behavioral tests. The frequency of Ca^2+^ events profoundly elevated during drug-seeking test after 30-day withdrawal, compared to drug-seeking test after 1-day withdrawal from cocaine-SA, but not Saline-SA (Fig. 1k–n and Supplementary Fig. S5a–c), suggesting an increase in the average neuronal activity of NAcSh cocaine-ensembles after prolonged abstinence. Furthermore, we found that nose poking of cocaine associated portal triggered comparable increases in GCaMP fluorescence in NAcSh cocaine-ensembles in WD1 and WD30 groups (Fig. 1o–q). The mean intensity of NAcSh GCaMP fluorescence showed no significant change after 30-day withdrawal (Supplementary Fig. S5d, e). These data are consistent with the increase of c-Fos expression in cocaine-ensembles induced by drug-seeking test after 30-day withdrawal.

### The increased activation of ensembles labeled by cocaine, not other neurons in NAcSh, mediates incubation of cocaine craving

**Fig. 2:**
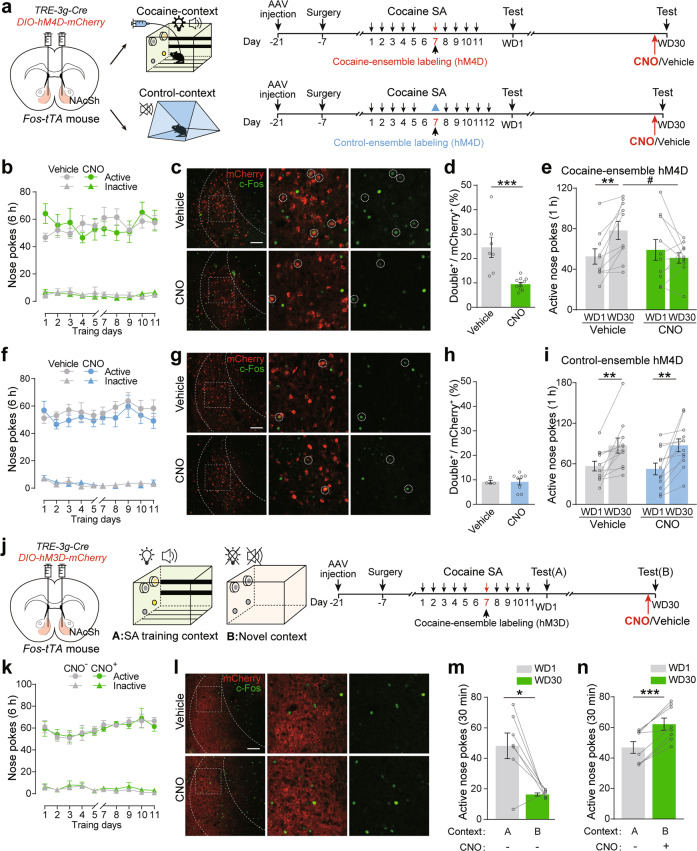
Activation of NAcSh cocaine-ensembles is required for incubation of cocaine craving. **a**–**i** *AAV*_*9*_*-TRE-3g-Cre* and *AAV*_*9*_*-hSyn-DIO-hM4D-mCherry* were injected in the NAcSh of *Fos-tTA* mice fed on a diet containing doxycycline (Dox, 40 mg/kg). Cocaine-SA training was performed on days 1–5 and 7–11/8–12. For NAcSh cocaine-ensemble labeling, a regular diet without Dox was provided on days 6–7 to allow *c-fos*-driven expression of hM4D-mCherry. For NAcSh control-ensemble labeling, a regular diet (without Dox) was provided on days 6–7 and the mice were exposed to a novel context without cocaine on day 7. Dox-containing diet (1 g/kg) was provided right after cocaine-SA training or a neutral context exposure on day 7. Drug-seeking tests (1 h) were performed after 1-day and 30-day withdrawal and mice were treated with CNO (1 mg/kg, i.p.) or saline (Vehicle, 4 ml/kg, i.p.) 30 min before tests after 30-day withdrawal. **a** Experimental scheme. **b** Plots of active and inactive nose pokes during cocaine-SA training. [Vehicle: *n* = 10, CNO: *n* = 10, Active nosepokes, *F*_group×session_ (9, 162) = 1.571, *p* = 0.128, Inactive nosepokes, *F*_group×session_ (9, 162) = 0.736, *p* = 0.675, RM two-way ANOVA]. Representative images (**c**) and bar graph of c-Fos^+^mCherry^+^/mCherry^+^ cells (**d**) in the NAcSh of *Fos-tTA* mice with expression of hM4D in NAcSh cocaine-ensembles treated with Vehicle or CNO. Red: mCherry, Green: c-Fos. Scale bar: 100 μm. [Vehicle *n* = 7, CNO *n* = 10, *U* = 2, *p* < 0.001, Mann–Whitney *U* test]. **e** Plots of active nose pokes in drug-seeking tests in the mice with expression of hM4D in NAcSh cocaine-ensembles. [Vehicle *n* = 10, CNO *n* = 10, *F*_treatment×session_ (1, 18) = 9.455, *p* = 0.007, RM two-way ANOVA]. **f** Plots of active and inactive nose pokes during cocaine-SA training. [Vehicle: *n* = 11, CNO: *n* = 12, Active nosepokes, *F*_group×session_ (9, 189) = 0.475, *p* = 0.890, Inactive nosepokes, *F*_group×session_ (9, 189) = 1.084, *p* = 0.376, RM two-way ANOVA]. Representative images (**g**) and bar graph of c-Fos^+^mCherry^+^/mCherry^+^ (%) (**h**) in the NAcSh of *Fos-tTA* mice with expression of hM4D in NAcSh control-ensembles treated with Vehicle or CNO. Red: mCherry, Green: c-Fos. Scale bar: 100 μm. [Vehicle *n* = 4, CNO *n* = 8, *t*_(10)_ = 0.027, *p* = 0.979, Two-tailed Student’s *t* test]. **i** Plots of active nose pokes in drug-seeking tests in the mice with expression of hM4D in NAcSh control-ensembles. [Veh *n* = 11, CNO *n* = 12, *F*_treatment×session_ (1, 21) = 0.119, *p* = 0.734, RM two-way ANOVA]. **j**–**n** *AAV-TRE-3g-Cre* and *AAV-hSyn-DIO-hM3D-mCherry* were injected in the NAcSh of *Fos-tTA* mice fed on diet containing Dox (40 mg/kg). Cocaine-SA training was performed on days 1–5 and 7–11. Regular diet without Dox was provided on days 6–7 to allow *c-fos*-driven expression of hM3D-mCherry. Dox-containing diet (1 g/kg) was provided right after cocaine-SA training on day 7. Drug-seeking tests (30 min) were performed in the SA training context (context A) after 1-day withdrawal and in a novel context (context B) after 30-day withdrawal. Mice were treated with CNO (1 mg/kg, i.p.) or vehicle (4 ml/kg, i.p.) 30 min before the tests after 30-day withdrawal. **j** Experimental scheme. **k** Plots of active and inactive nose pokes during cocaine-SA training. [CNO^−^: *n* = 7, CNO^+^: *n* = 8, Active nosepokes, *F*_group×session_ (9, 117) = 0.231, *p* = 0.989, Inactive nosepokes, *F*_group×session_ (9, 117) = 0.189, *p* = 0.995, RM two-way ANOVA]. **l** Representative images in the NAcSh of *Fos-tTA* mice with expression of hM3D in NAcSh cocaine-ensembles treated with Vehicle or CNO. **m**, **n** Plots of active nose pokes in drug-seeking tests. [Vehicle group: *n* = 7, *t*_(6)_ = 3.552, *p* = 0.012; CNO group: *n* = 8, *t*_(7)_ = 6.978, *p* < 0.001, Two-tailed paired *t*-test]. *p < 0.05, **p < 0.01, ***p < 0.001, and ^#^*p* < 0.05 vs. indicated group.

To examine the roles of NAcSh cocaine-ensembles in incubation for cocaine craving, we expressed hM4D-mCherry in cocaine-ensembles in the NAcSh of *Fos:tTA* mice and selectively silenced NAcSh cocaine-ensembles by injection of clozapine-N-oxide (CNO, 1 mg/kg, i.p.) (Fig. 2a). CNO treatment significantly decreased c-Fos expression and excitability in NAcSh cocaine-ensembles (Fig. 2b–d and Supplementary Fig. S6d–f). Chemogenetic inhibition of NAcSh cocaine-ensembles significantly decreased the active nose pokes during drug-seeking test after 30-day withdrawal (Fig. 2e), suggesting chemogenetic inhibition of NAcSh cocaine-ensembles suppresses incubation of cocaine craving. To examine the specificity of the effect of NAcSh ensembles on cocaine craving, we expressed hM4D-mCherry in NAcSh ensembles activated by a non-cocaine-related neutral context (control-ensembles). The number of control-ensembles was similar to cocaine-ensembles in the NAcSh (Supplementary Fig. S6a–c). Silencing the control-ensembles had no significant effects on neuronal activation in the NAcSh and the active nose pokes after 30-day withdrawal (Fig. 2f–i). These data suggest that activation of NAcSh ensembles recruited during cocaine-SA training is required for incubation of cocaine craving.

Next, we expressed hM3D-mCherry in NAcSh cocaine-ensembles and tested the effect of activation of these ensembles on cocaine-seeking behavior. CNO treatment significantly increased c-Fos expression and excitability in NAcSh cocaine-ensembles (Fig. 2j–l and Supplementary Fig. S6g–l). Omission of drug-associated cues, including tone, light, and context, attenuated the induction of cocaine-seeking behavior, while chemogenetic activation of NAcSh cocaine-ensembles after 30-day withdrawal rescued the impaired induction of active nose pokes for cocaine and increased active nose pokes further (Fig. 2j–n). These data suggest that the increased activation or susceptibility to activation of NAcSh neuronal ensembles mediates incubation of cocaine craving.

### Prolonged withdrawal increases membrane excitability of cocaine-ensembles

**Fig. 3:**
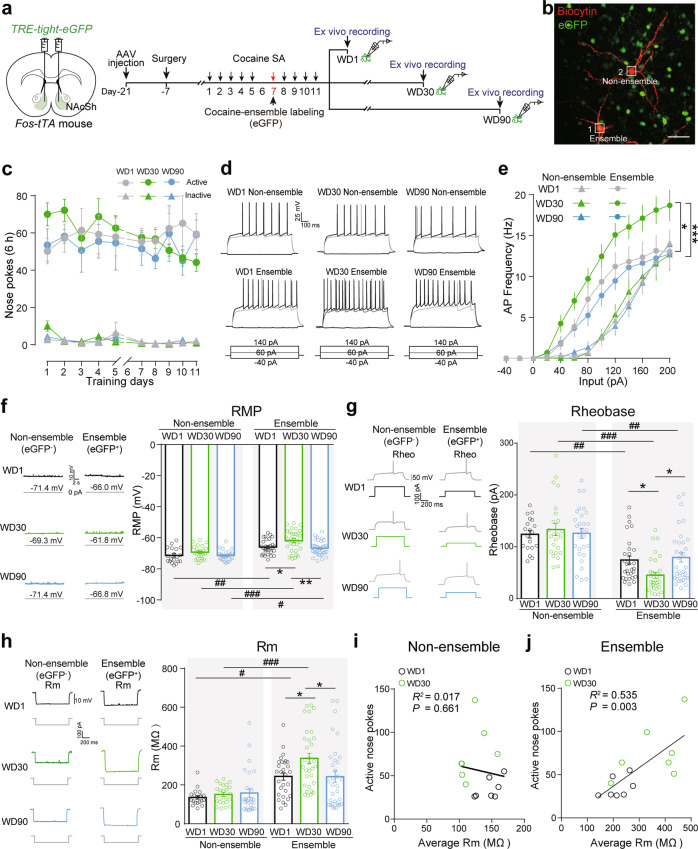
The membrane excitability of NAcSh cocaine-ensembles is enhanced after prolonged withdrawal. **a**–**j** *AAV-TRE-tight-eGFP* was injected in the NAcSh of *Fos-tTA* mice fed on diet containing Dox (40 mg/kg). Regular diet without Dox was provided on days 6–7 and cocaine-ensembles were labeled in SA training on day 7. The ex vivo recordings were performed after 1-day, 30-day, and 90-day withdrawal without seeking test. **a** Experimental scheme. **b** Representative images of biocytin-filled ensemble and non-ensemble in the NAcSh. Red: Biocytin, Green: eGFP. Scale bar: 100 μm. **c** Plots of active and inactive nose pokes during cocaine-SA training. [WD1: *n* = 5, WD30: *n* = 5, WD90: *n* = 6, Active nosepokes, *F*_group×session_ (18, 117) = 1.079, *p* = 0.382, Inactive nosepokes, *F*_group×session_ (18, 117) = 1.391, *p* = 0.149, RM Two-way ANOVA]. Representative AP traces (**d**) and graphs of AP frequency (**e**) at the indicated current steps in NAcSh cocaine non-ensembles and ensembles [non-ensemble: WD1 *n* = 20 cells/5 mice, WD30 *n* = 24 cells/5 mice, WD90 *n* = 28 cells/6 mice, WD1 vs. WD30*, F*_group×session_ (12,504) = 0.259, *p* = 0.995; WD1 vs. WD90, *F*_group×session_ (12,552) = 0.102, *p* = 0.999; WD30 vs. WD90, *F*_group×session_ (12,600) = 0.511, *p* = 0.909; ensemble: WD1 *n* = 22 cells/5 mice, WD30 *n* = 33 cells/5 mice, WD90 *n* = 30 cells/6 mice, WD1 vs. WD30, *F*_group×session_ (12,636) = 1.901, *p* = 0.031; WD1 vs. WD90, *F*_group×session_ (12,600) = 0.273, *p* = 0.993; WD30 vs. WD90, *F*_group×session_ (12,732) = 3.335, *p* < 0.001, RM Two-way ANOVA]. Representative trace and graphs of the resting membrane potential (RMP) (**f**), rheobase (**g**), and membrane resistance (Rm) (**h**) of NAcSh cocaine non-ensembles and ensembles. [WD1 non-ensemble *n* = 19 cells/5 mice, WD30 non-ensemble *n* = 24 cells/5 mice, WD 90 non-ensembles *n* = 28 cells/6 mice, WD1 ensemble *n* = 29 cells/5 mice, WD30 ensemble *n* = 32 cells/5 mice, WD90 ensemble *n* = 33 cells/6 mice, RMP: *F*_cell type×session_ (2, 159) = 1.313, *p* = 0.272; Rheobase: *F*_cell type×session_ (2, 159) = 3.571, *p* = 0.030; Rm: *F*_cell type×session_ (1, 159) = 2.788, *p* = 0.065, Two-way ANOVA]. Graph of correlation between the average Rm of recorded cocaine non-ensembles (**i**) or ensembles (**j**) and active nose pokes during test [WD1: *n* = 7, WD30: *n* = 7, Non-ensemble: *R*^2^ = 0.017, *p* = 0.661; Ensemble: *R*^2^ = 0.535, *p* = 0.003]. *p < 0.05, **p < 0.01, ***p < 0.001, ^#^*p* < 0.05, ^##^*p* < 0^.^01, ^###^*p* < 0.001 vs. indicated group.

**Fig. 4:**
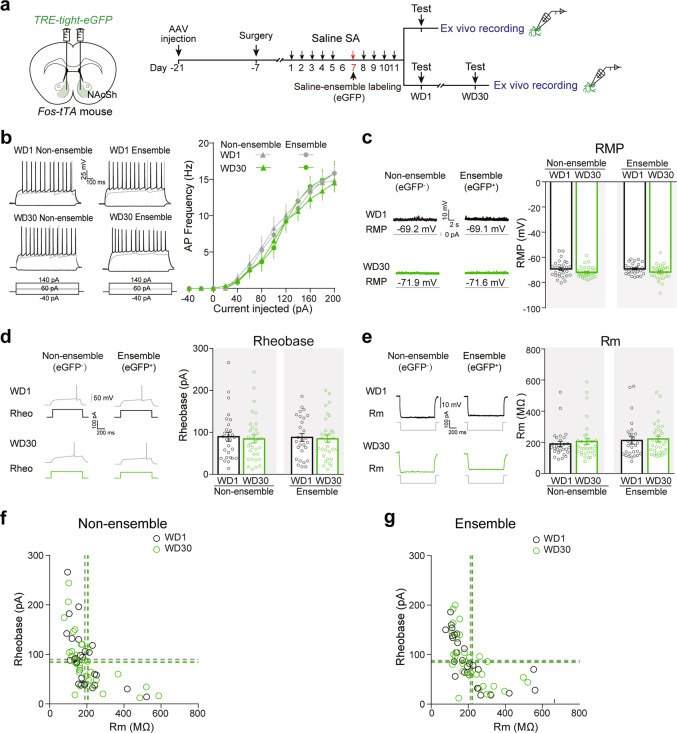
Membrane excitability of NAcSh saline-ensembles does not change after prolonged withdrawal. **a**–**g** *AAV-TRE-tight-eGFP* was injected in the NAcSh of *Fos-tTA* mice fed on diet containing Dox (40 mg/kg). Saline-SA training was performed on days 1–5 and 7–11. Saline-ensembles were labeled on day 7. The ex vivo recordings were performed after drug-seeking tests after 1-day and 30-day withdrawal. **a** Experimental scheme. **b** Representative AP traces and graph of AP frequency at the indicated current steps in saline non-ensembles and ensembles [non-ensemble: WD1 *n* = 22 cells/6 mice, WD30 *n* = 26 cells/7 mice, *F*_session×current_ (12, 552) = 0.373, *p* = 0.973; ensemble: WD1 *n* = 25 cells/6 mice, WD30 *n* = 26 cells/7 mice, *F*_session×current_ (12, 588) = 0.388, *p* = 0.968, RM Two-way ANOVA]. Representative trace and graph of RMP (**c**), rheobase (**d**), and Rm (**e**) in saline non-ensembles and ensembles. [RMP: WD1 Non-ensemble *n* = 28 cells/6 mice; WD30 Non-ensemble *n* = 33 cells/7 mice; WD1 ensemble: *n* = 28 cells/6 mice; WD30 ensemble: *n* = 32 cells/7 mice, *F*_cell type×session_ (1, 117) = 0.009, *p* = 0.926; rheobase: WD1 Non-ensemble *n* = 28 cells/6 mice, WD30 Non-ensemble *n* = 33 cells/7 mice, WD1 ensemble *n* = 28 cells/6 mice, WD30 ensemble *n* = 32 cells/7 mice, *F*_cell type×session_ (1, 117) = 0.013, *p* = 0.911; Rm: WD1 Non-ensemble *n* = 28 cells/6 mice, WD30 Non-ensemble *n* = 33 cells/7 mice, WD1 ensemble *n* = 28 cells/6 mice, WD30 ensemble *n* = 32 cells/7 mice, *F*_cell type×session_(1, 117) = 0.024, *p* = 0.878, Two-way ANOVA]. Distribution of the rheobase and Rm of saline non-ensembles (**f**) and ensembles (**g**). [WD1 Non-ensemble *n* = 28 cells/6 mice, WD30 Non-ensemble *n* = 33 cells/7 mice, Rheobase, *t*_(59)_ = 0.371, *p* = 0.712; Rm, *t*_(59)_ = 0.566, *p* = 0.574; WD1 ensemble *n* = 28 cells/6 mice, WD30 ensemble *n* = 32 cells/7 mice, Rheobase, *t*_(58)_ = 0.242, *p* = 0.810; Rm, *t*_(58)_ = 0.318, *p* = 0.752, Two-tailed Student’s *t* test].

To evaluate the membrane excitability of cocaine-ensembles and surrounding non-ensembles after prolonged withdrawal, we performed ex vivo electrophysiology recordings on 1-day, 30-day and 90-day withdrawal (Fig. 3a–c). The action potential (AP) frequency of cocaine-ensembles (eGFP^+^), but not cocaine-non-ensembles (eGFP^−^), was significantly increased after 30-day withdrawal (Fig. 3d, e). Significant enhancement of RMP was observed in NAcSh cocaine-ensembles after 1-day and 30-day withdrawal, and 30-day withdrawal increased RMP further (Fig. 3f). Consistent with the increase of the excitability of NAcSh cocaine-ensembles after prolonged withdrawal, we also detected a significant reduction of rheobase and increase of membrane input resistance in NAcSh ensembles, but not in non-ensembles after 30-day withdrawal (Fig. 3g, h). After 90-day withdrawal, AP frequency, RMP, rheobase, and membrane input resistance of NAcSh ensembles returned to a level comparable to WD1 (Fig. 3d–h). Similar results were obtained when we examined the membrane excitability of ensembles and non-ensembles after drug-seeking tests (Supplementary Fig. S7), indicating that cue-induced drug-seeking test does not affect membrane excitability. The AP latency, the time from onset of rheobase current step to AP threshold, and half-width of AP were selectively decreased in NAcSh cocaine-ensembles (Supplementary Table S1). In addition, the number of active nose pokes of each mouse at WD1-Test or WD30-Test was positively correlated with membrane input resistance of ensembles, but not non-ensembles (Fig. 3i, j and Supplementary Fig. S7). The AP frequency, RMP, rheobase, and membrane input resistance of NAcSh ensembles labeled by saline-SA training (saline-ensemble) did not change after 30-day withdrawal (Fig. 4 and Supplementary Table S2). These results support the notion that prolonged withdrawal selectively increases membrane excitability of NAcSh cocaine-ensembles, which may facilitate cocaine-ensemble activation and incubated cocaine-seeking after abstinence.

### Prolonged withdrawal downregulates ensemble IRK currents, increases ensemble excitability, and facilitates incubated cocaine-seeking

**Fig. 5:**
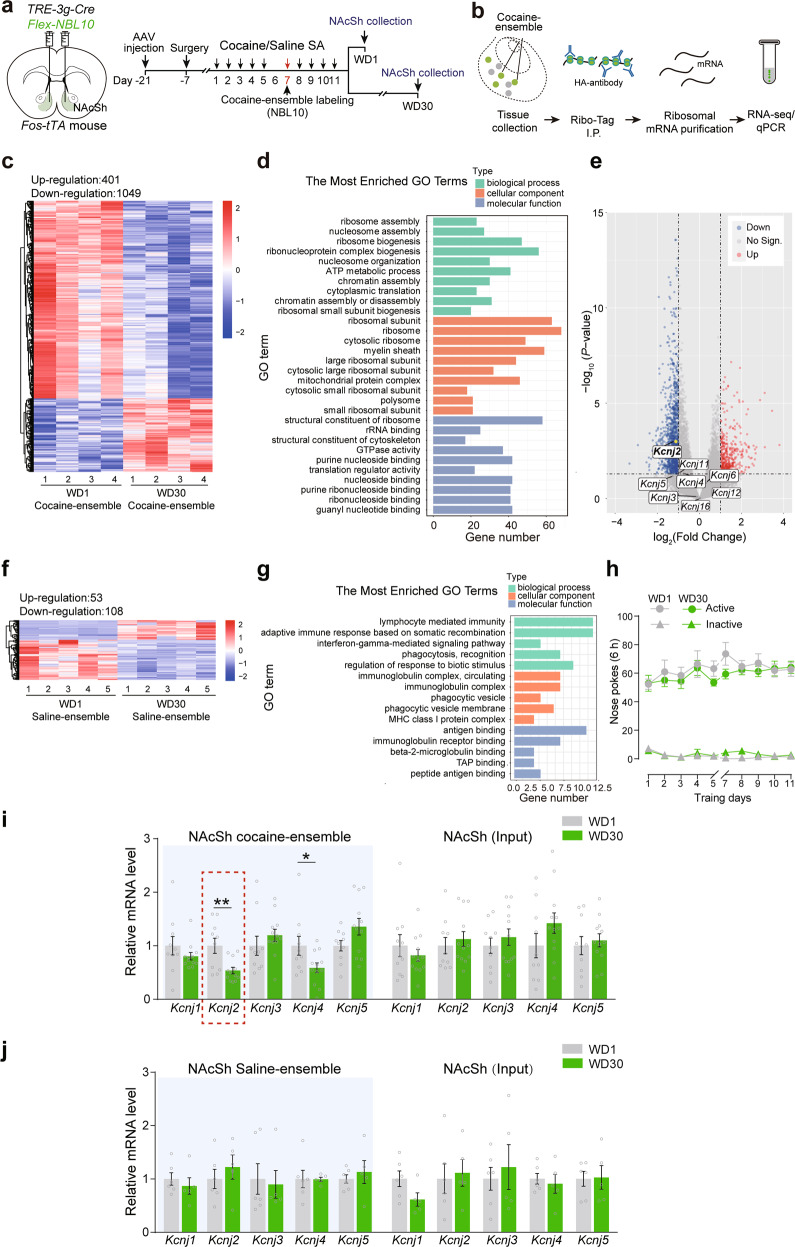
Kir_2.1_ expression in NAcSh cocaine-ensembles is decreased after prolonged cocaine withdrawal. **a**–**j** *AAV-TRE-3g-Cre* and *AAV-FLEX-NBL10* were injected in the NAcSh of *Fos-tTA* mice fed on a diet containing Dox (40 mg/kg). Cocaine-ensembles and saline-ensembles were labeled, and the mouse brain sections containing the NAcSh were dissected and collected after 1-day and 30-day withdrawal for ribosomal mRNA purification. **a**, **b** Experimental scheme. **c**, **f** Heatmap demonstrating the expression of differentially expressed genes (fold change >2 or <0.5 and *p* value <0.05) in NAcSh cocaine-ensembles and saline-ensembles of WD30 groups, compared with WD1 groups. **d**, **g** The most enriched GO term of differentially expressed genes of cocaine-ensembles and saline-ensembles in WD30, compared with WD1. **e** Volcano plot showing ribosome-associated transcripts with at least two-fold differential expression in NAcSh cocaine-ensembles in WD30 group, compared with WD1. Genes that encode IRK family were labeled. *Kcnj2* was marked with a yellow dot. **h** Plots of active and inactive nose pokes during cocaine-SA training. [WD1 group: *n* = 10, WD30 group: *n* = 12, Active nosepokes, *F*_group×session_ (9, 180) = 0_._713, *p* = 0.696, Inactive nosepokes, *F*_group×session_ (9, 180) = 1_._518, *p* = 0.145, RM two-way ANOVA]. **i**, **j** Quantification of relative mRNA levels of IRK family members in NAcSh cocaine-ensembles, saline-ensembles, and total RNA. [NAcSh cocaine-ensemble: WD1 *n* = 10, WD30 *n* = 12, *Kcnj2*, *U* = 19, *p* = 0.005, Mann–Whitney *U* test; *Kcnj4*: *t*_(20)_ = 2.183, *p* = 0.041, Two-tailed Student’s *t* test; NAcSh saline*-*ensembles: WD1 *n* = 6, WD30 *n* = 5, *Kcnj2*, *t*_(9)_ = 0.782, *p* = 0.454, Two-tailed Student’s *t* test]. *p < 0.05, and **p < 0^.^01 vs. indicated group.

We isolated and sequenced ribosome-associated (actively translated) mRNAs from the NAcSh cocaine-ensembles by Shannon entropy-based method and ANOVA revealed significant changes between WD1 and WD30 groups (Fig. 5a, b). Gene ontology analysis of differentially expressed genes among cocaine-ensembles revealed significant enrichment of genes in the various pathways, especially ribosomal assembly, ribosomal subunit, ATP metabolic process, GTP activity, etc. (Fig. 5c, d). To access transcription-dependent regulation of neuronal activity, we mined RNA-seq data for K^+^ channel genes that may underlie functional differences. We found the expression of a variety of IRK family genes was changed, especially *Kcnj2* (potassium inwardly rectifying channel subfamily J member 2). The mRNA of *Kcnj2* was significantly downregulated after 30-day withdrawal in NAcSh cocaine-ensembles, but not in NAcSh saline-ensembles (Fig. 5c–g). We verified the above results by real-time PCR with ribosome-associated mRNAs from the NAcSh cocaine-ensembles. Consistently, we found that *Kcnj2* transcripts were significantly decreased in the NAcSh cocaine-ensembles after 30-day withdrawal, but not in saline-ensembles, and after 90-day withdrawal, the *Kcnj2* mRNA returned to the levels of WD1 group (Fig. 5i, j and Supplementary Fig. S8a–c). K_v_, K_Ca_, and K2P families are also key determinants of membrane excitability [18]. The mRNA level of of K_v_, K_Ca_, and K2P family members tested was not significantly changed in the NAcSh cocaine-ensembles after 30-day withdrawal from cocaine-SA (Supplementary Fig. S8d, e).

**Fig. 6:**
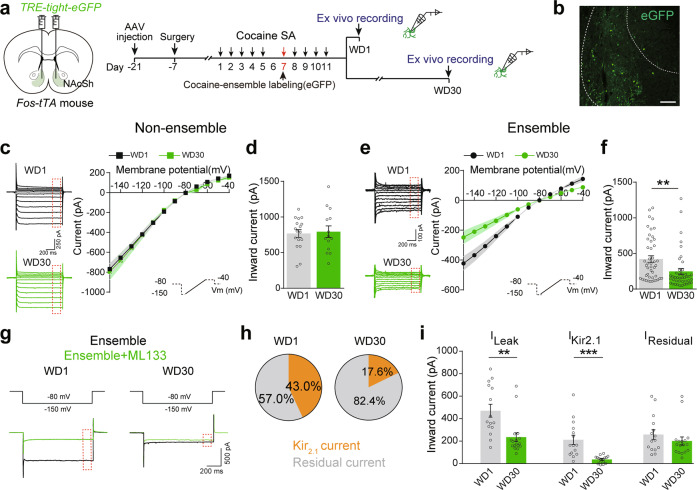
Kir_2.1_ currents in NAcSh cocaine-ensembles are decreased after prolonged cocaine withdrawal. **a**–**i** *AAV-TRE-tight-eGFP* was injected in the NAcSh of *Fos-tTA* mice fed on a diet containing Dox (40 mg/kg). Cocaine-ensembles were labeled, and the ex vivo recordings were performed after 1-day and 30-day withdrawal. **a** Experimental scheme. **b** Representative image of eGFP expression in NAcSh ensembles. Green: eGFP. Scale bar, 100 μm. Representative trace and graph of IRK currents in NAcSh cocaine non-ensembles (**c**) and ensembles (**e**). [Non-ensemble: WD1 *n* = 17 cells/6 mice, WD30 *n* = 14 cells/8 mice, *F*_session×voltage_ (11, 319) = 0.157, *p* = 0.999; Ensemble: WD1 *n* = 46 cells/9 mice, WD30 *n* = 42 cells/9 mice, *F*_session×voltage_ (11, 946) = 9.488, *p* < 0.001, RM Two-way ANOVA]. **d**, **f** Bar graph of inward currents in NAcSh cocaine non-ensembles and ensembles [Non-ensemble: WD1 *n* = 17 cells/6 mice, WD30 *n* = 14 cells/8 mice, *t*_(29)_ = 0.269, *p* = 0.790; Ensemble: WD1 *n* = 46 cells/9 mice, WD30 *n* = 42 cells/9 mice, *t*_(86)_ = 2.826, *p* = 0.006, Two-tailed Student’s *t* test]. Representative trace (**g**) and percentage of leaky current sensitive to ML133 (50 μM) and residual IRK currents of NAcSh ensembles (**h**). **i** Bar graph of IRK currents, Kir_2.1_ currents, and the residual IRK currents of NAcSh ensembles. [WD1 *n* = 15 cells/8 mice, WD30 *n* = 17 cells/6 mice, I_leak_: *t*_(30)_ = 3.457, *p* = 0.002, Two-tailed Student’s *t* test; I_Kir2.1_: *U* = 16, *p* < 0.001, Mann–Whitney *U* test; I_residual_: *t*_(30)_ = 1.015, *p* = 0.318, Two-tailed Student’s *t* test]. **p < 0.01, and ***p < 0.001 vs. indicated group.

The inward rectifying potassium (IRK) channels regulate membrane excitability through their influence on membrane resistance [29]. The immunostaining data showed that membrane Kir_2.1_ expression was decreased after 30-day withdrawal specifically in NAcSh ensembles, but not non-ensembles, and returned after 90-day withdrawal (Supplementary Fig. S8f–h). Then we measured IRK currents that are sensitive to Cs^+^ [30, 31]. Cesium chloride (CsCl) significantly decreased currents elicited by hyperpolarizing in NAcSh D1-MSNs (Supplementary Fig. S8i–m). Thus, the inward current evoked with the voltage clamp protocol was used as a proxy for IRK currents without CsCl subtraction. The result of voltage-clamp recording showed IRK currents were decreased in NAcSh ensembles, but not in non-ensembles, after 30-day withdrawal (Fig. 6a–f). Bath application of ML133, a Kir_2.1_ blocker, suppressed 43.0% of total IRK current in NAcSh ensembles after 1-day withdrawal and suppressed 17.6% of total IRK current in ensembles after 30-day withdrawal, suggesting that prolonged withdrawal significantly decreased leaky current through the reduction of Kir_2.1_ currents in NAcSh ensembles (Fig. 6g–i).

**Fig. 7:**
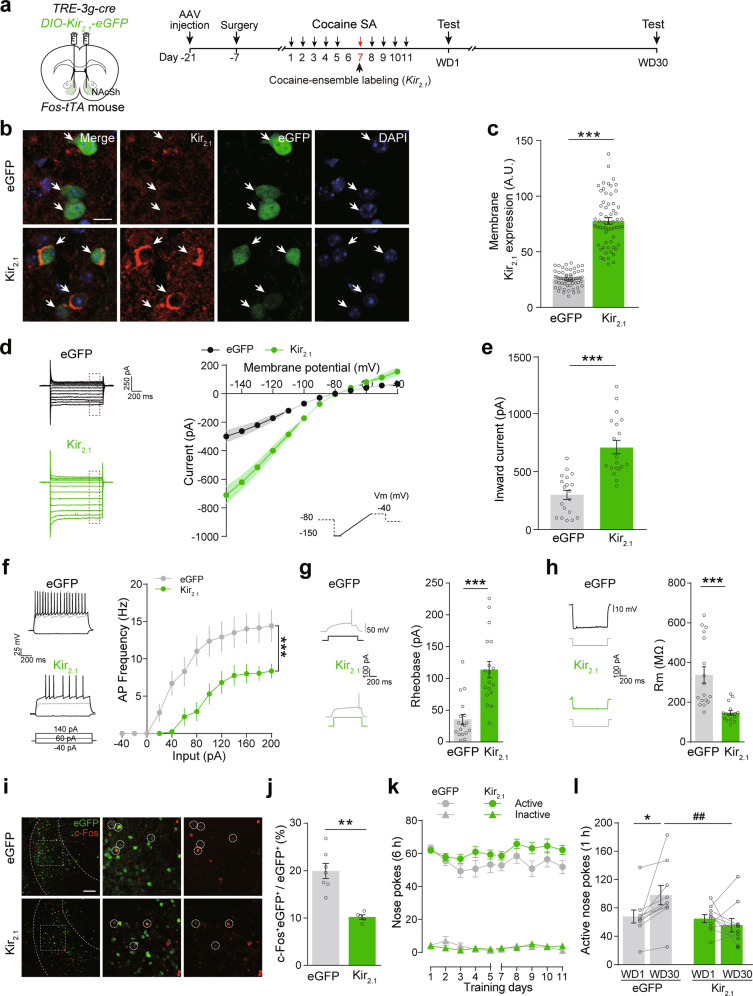
Upregulation of Kir_2.1_ membrane expression and currents in NAcSh cocaine-ensembles inhibits incubation of cocaine craving. **a**–**l** *AAV-TRE-3g-Cre* and *AAV-DIO-Kir*_*2.1*_*-eGFP* were injected in the NAcSh of *Fos-tTA* mice and cocaine-ensembles were labeled during cocaine-SA training. Drug-seeking tests (1 h) were performed after 1-day and 30-day withdrawal. Mice were sacrificed for ex vivo recordings or IHC 60 min after tests after 30-day withdrawal. **a** Experimental scheme. Representative images (**b**) and bar graph of quantification of Kir_2.1_ membrane expression (**c**) in NAcSh ensembles (eGFP^+^). Green: eGFP, Red: Kir_2.1_, Blue: DAPI. Scale bar: 50 μm. [eGFP *n* = 60 cells/3 mice, Kir_2.1_ *n* = 61 cells/3 mice, *U* = 1, *p* < 0.001, Mann–Whitney *U* test]. **d**, **e** Representative trace and graphs of IRK currents of NAcSh ensembles. [eGFP *n* = 19 cells/4 mice, Kir_2.1_ *n* = 19 cells/4 mice, **d**: *F*_virus×voltage_ (11, 396) = 32.408, *p* < 0.001, RM Two-way ANOVA; **e**: *U* = 23, *p* < 0.001, Mann–Whitney *U* test]. **f** Representative trace and graph of AP spike frequency at the indicated current steps of NAcSh ensembles. [eGFP *n* = 14 cells/4 mice, Kir_2.1_ *n* = 17 cells/4 mice *F*_virus×current_ (11, 348) = 3.989, *p* < 0.001, RM Two-way ANOVA]. Representative trace and graphs of rheobase (**g**) and Rm (**h**) of NAcSh ensembles. [Rheobase: eGFP *n* = 18 cells/4 mice, Kir_2.1_ *n* = 18 cells/4 mice, *U* = 28, *p* < 0.001; Rm: eGFP *n* = 17 cells/4 mice, Kir_2.1_ *n* = 16 cells/4 mice, *U* = 23, *p* < 0.001, Mann–Whitney *U* test]. Representative images (**i**) and quantification of c-Fos^+^eGFP^+^/eGFP^+^ ratio (%) (**j**). Green: eGFP, Red^:^ c-Fos. Scale bar: 100 μm. [eGFP *n* = 7, Kir_2.1_ *n* = 5, *U* = 0, *p* = 0.003, Mann–Whitney *U* test]. **k** Plots of active and inactive nose pokes during cocaine-SA training. [eGFP: *n* = 10, Kir_2.1_: *n* = 10, Active nosepokes, *F*_virus×session_ (9, 162) = 1.149, *p* = 0.332, Inactive nosepokes, *F*_virus×session_ (9, 162) = 1.414, *p* = 0.186, RM two_-_way ANOVA]. **l** Plots of active nose pokes in drug-seeking tests after 1-day and 30-day withdrawal. [eGFP *n* = 10, Kir_2.1_ *n* = 10, *F*_virus×session_ (1, 18) = 6.883, *p* = 0.017, RM Two-way ANOVA]. *p < 0.05, **p < 0.01, ***p < 0.001, and ^##^*p* < 0.01 vs. indicated group.

**Fig. 8:**
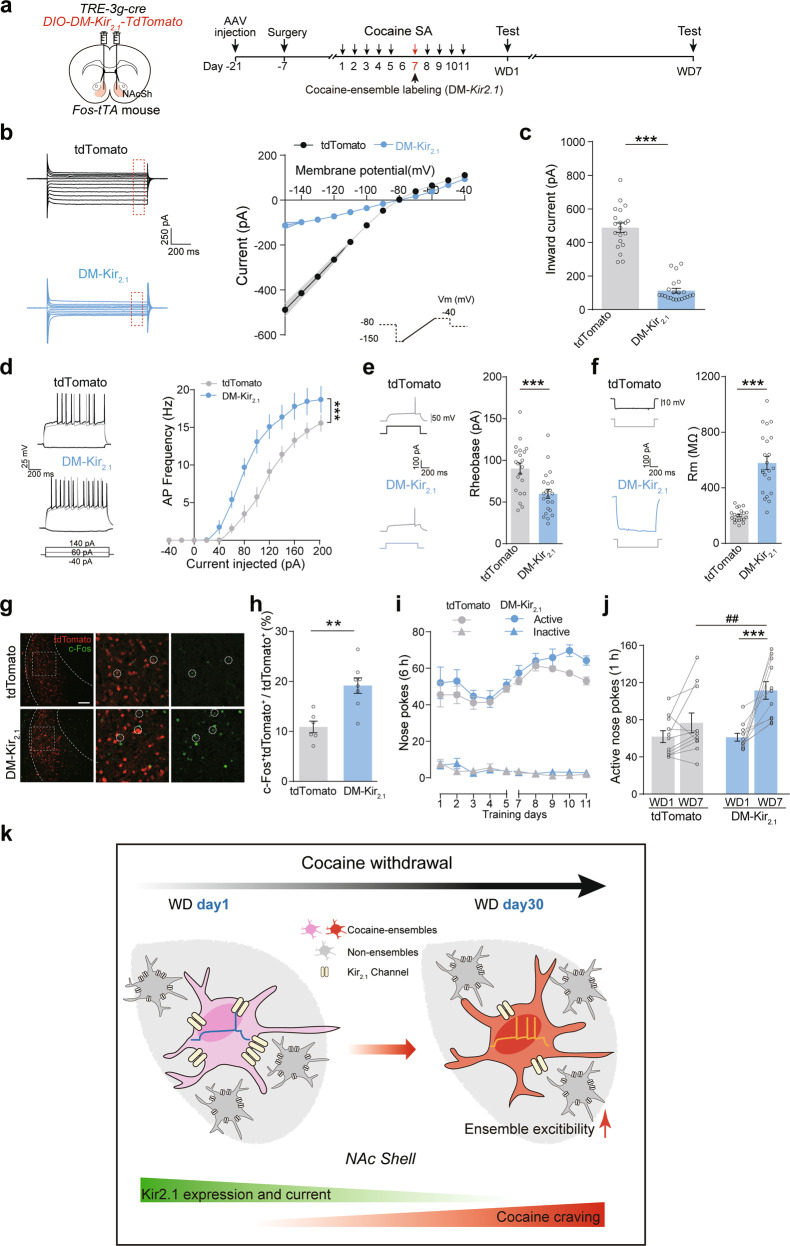
Expression of dominant-negative mutant of Kir_2.1_ in NAcSh cocaine-ensembles decreases Kir_2.1_ currents and promotes incubation of cocaine craving. **a**–**j** *AAV-TRE-3g-Cre* and *AAV-DIO-DM-Kir*_*2.1*_*-tdTomato* were infected in the NAcSh of *Fos-tTA* mice, and cocaine-ensembles were labeled during cocaine-SA training. Drug-seeking tests (1 h) were performed after 1-day and 7-day withdrawal. Mice were sacrificed for ex vivo recordings or IHC 60 min after tests after 7-day withdrawal. **a** Experimental scheme. **b**, **c** Representative trace and graphs of IRK currents of NAcSh ensembles. [tdTomato *n* = 20 cells/3 mice, DM-Kir_2.1_ *n* = 22 cells/3 mice, **b**: *F*_virus×voltage_ (11, 440) = 112.699, *p* < 0.001, RM Two-way ANOVA; **c**: *U* = 0, *p* < 0.001, Mann–Whitney *U* test]. **d** Representative trace and graph of AP frequency at the indicated current steps of NAcSh ensembles. [tdTomato *n* = 21 cells/3 mice, DM-Kir_2.1_ *n* = 20 cells/3 mice, *F*_virus×current_ (12, 468) = 4.905, *p* < 0.001, RM Two-way ANOVA]. Representative trace and graphs of rheobase (**e**) and Rm (**f**) of NAcSh ensembles. [tdTomato *n* = 21 cells/3 mice, DM-Kir_2.1_ *n* = 22 cells/3 mice, Rheobase: *t*_(41)_ = 3.571, *p* = 0.001, Two-tailed Student’s *t* test. Rm: *U* = 5, *p* < 0.001, Mann–Whitney *U* test]. Representative images (**g**) and quantification of c-Fos^+^tdTomato^+^/tdTomato^+^ ratio (%) (**h**). Red: tdTomato, Green: c-Fos. Scale bar: 100 μm. [tdTomato: *n* = 6, DM-Kir_2.1_ *n* = 8, *t*_(12)_ = 4.003, *p* = 0.002, Two-tailed Student’s *t* test]. **i** Plots of ac*t*ive and **i**nactive nose pokes during cocaine-SA training. [tdTomato: *n* = 11, DM-Kir_2.1_: *n* = 11, Active nosepokes, *F*_virus×session_ (9, 180) = 0_._526, *p* = 0.854, Inactive nosepokes, *F*_virus×session_ (9, 180) = 1_._141, *p* = 0.336, RM two-way ANOVA]. **j** Plots of active nose pokes in drug-seeking tests after 1-day and 7-day withdrawal. [tdTomato *n* = 11, DM-Kir_2.1_ *n* = 11, *F*_virus×session_ (1, 20) = 6.626, *p* = 0.018, RM Two-way ANOVA]. **p < 0.01, ***p < 0.001 and ^##^*p* < 0.01 vs. indicated group. **k** Working model illustrates that Kir_2.1_ expression and currents decrease specifically in NAcSh cocaine-ensembles after prolonged withdrawal, which increases the membrane excitability of NAcSh cocaine-ensembles and promotes incubation for cocaine craving.

To further address the functional contribution of Kir_2.1_ expression to incubation of cocaine craving, we tested the effects of overexpression of wild type Kir_2.1_ or a mutant-pore Kir_2.1_ as a dominant-negative Kir_2.1_ (DM-Kir_2.1_) [32] in NAcSh ensembles on cocaine-seeking behavior. Expression of Kir_2.1_ significantly increased IRK current and rheobase and decreased the AP frequency and membrane input resistance in NAcSh cocaine-ensembles after 30-day withdrawal (Fig. 7a–h). The expression of Kir_2.1_ in NAcSh ensembles significantly inhibited ensemble activation (Fig. 7i, j) and reduced the active nose pokes after 30-day withdrawal (Fig. 7k, l), but did not change locomotor activity and anxiety level (Supplementary Fig. S9a–c). In contrast, expression of DM-Kir_2.1_ significantly decreased IRK current and rheobase and increased the AP frequency and membrane input resistance in NAcSh cocaine-ensembles after 7-day withdrawal (Fig. 8a–f). The expression of DM-Kir_2.1_ in NAcSh ensembles significantly increased ensemble activation and the active nose pokes after 7-day withdrawal (Fig. 8g–j), without influence on locomotor activity and anxiety level (Supplementary Fig. S9d–f), indicating an accelerated incubation of cocaine craving. These data suggest that prolonged withdrawal leads to downregulation of *Kcnj2* expression and dysfunction of Kir_2.1_ channels specifically in the NAcSh cocaine-ensembles and thus increases membrane excitability of cocaine-ensembles, facilitating the induction of drug-seeking behavior upon exposure to conditional stimuli.

## Discussion

Our results provide evidence that intrinsic excitability of accumbens neuronal ensembles activated by cocaine exposure is increased during withdrawal from cocaine, which promotes incubation of cocaine craving. As illustrated in Fig. 8k, we propose an updated working model about how NAcSh cocaine-ensembles contribute to incubation for cocaine craving. The increased activation of NAcSh cocaine-ensembles is induced by cue-induced drug-seeking after prolonged withdrawal of cocaine. The activation of NAcSh cocaine-ensembles is positively correlated with the degree of cue-induced craving for cocaine. After the prolonged withdrawal, the membrane excitability is significantly increased and IRK current is decreased specifically in NAcSh cocaine-ensembles. *Kcnj2* translation and Kir_2.1_ membrane expression are decreased specifically in NAcSh cocaine-ensembles after prolonged withdrawal. Expression of Kir_2.1_ in NAcSh cocaine-ensembles restores membrane excitability and then suppresses incubation for cocaine craving, while dysfunction of Kir_2.1_ by DM-Kir_2.1_ expression in NAcSh cocaine-ensembles accelerate incubation for cocaine craving. Our study suggests that prolonged withdrawal increases the excitability of NAcSh cocaine-ensembles via downregulation of Kir_2.1_ expression and currents, which promotes incubation of cocaine craving.

### Critical roles of ensembles activated by initial cocaine exposure in incubation of cocaine craving

Recent studies show that the neuronal ensembles activated by the presentation of drug cues after voluntary abstinence are critical for the expression of incubation of drug craving. Inactivation of cue-activated neuronal ensembles labeled after 12-day withdrawal in the central amygdala suppresses nicotine craving [21]; inactivation of cue-activated neurons in the dorsolateral striatum after 18-day withdrawal inhibits methamphetamine seeking [23]; inactivation of cue-activated neurons after 12-day withdrawal in the orbitofrontal cortex decreases heroin craving [22]. How neuronal ensembles tagged by initial drug exposure contribute to incubation of drug craving is unclear. Neuronal ensembles with elevated CREB expression in the lateral amygdala recruited during cocaine conditioning are critical for the expression of cocaine conditioned place preference (CPP) [33]. Our previous study suggests that activation of NAccore and vCA1 ensembles tagged during cocaine conditioning is required for cocaine CPP memory retrieval [26]. These findings indicate that specific neuronal ensembles tagged during initial cocaine exposure are possibly involved in drug addiction [34]. In this study, we speculate that NAcSh neuronal ensembles recruited by cocaine-SA might endure neuroadaptations during prolonged withdrawal and promote increasing craving for cocaine after abstinence. In addition, the relationship between memory and drug addiction has been put forward. Ensembles of the instrumental association potentiated by the conditioned reinforcing properties of the drug may mediate cue-induced craving [35]. It is possible that the stronger cocaine memory will be developed after prolonged abstinence and this enhanced memory will also lead to increased nose pokes. The previous study shows that VTA ensembles activated by morphine exposure preferentially project to the NAc, and induce dopamine-dependent reward and positive reinforcement. Disinhibition of VTA morphine ensemble alleviates the negative effects during opioid withdrawal [36]. Thus, we speculate that NAcSh cocaine-ensemble labeled during cocaine SA-training primarily encodes reward effects of cocaine, rather than active operating behavior. Prolonged withdrawal may increase craving for cocaine, not active operating behavior, through upregulation of NAcSh cocaine-ensemble excitability. Whether the NAcSh cocaine-ensembles encode stronger memory or increased craving for cocaine after abstinence needs further investigation.

The NAc plays a major role in incubation of drug craving [7, 37, 38]. It is thought to facilitate drug seeking by integrating dopaminergic and glutamatergic innervation and plasticity in the glutamatergic synapses on medium spiny neurons (MSNs) is extremely vulnerable to drugs of abuse [39–42]. Prominent glutamate inputs to the NAc from the ventral hippocampus (vHipp), basolateral amygdala (BLA), and medial prefrontal cortex (mPFC) produce divergent responses in cocaine exposure [43]. Withdrawal from cocaine-SA evokes an increase of AMPA/NMDA ratio at vHipp to D1-MSNs, but a decrease of AMPA/NMDA ratio and induction of CP-AMPAR insertion at mPFC to D1-MSNs [44]. Synaptic insertion of CP-AMPARs at BLA-NAcSh synapses during the withdrawal from cocaine-SA promotes incubation of cocaine craving [45]. Reversal of CP-AMPAR insertion at the infralimbic prefrontal cortex (IL)-to-NAcSh and non-CP-AMPAR insertion at the prelimbic prefrontal cortex (PrL)-to-NAccore projections potentiates and inhibits incubation of cocaine craving, respectively [46]. After a drug-free period, spatial and temporal representations in NAc projecting IL neuronal activity are reduced and activation of IL-NAc projection inhibits incubation of cocaine craving [47]. In addition, inhibition of IL paired with unreinforced lever presses prevents extinction learning and increases cocaine-seeking in reinstatement [48]. Among these glutamatergic projections, the IL mediates the extinction of drug seeking and IL-NAc projection is thought to inhibit cocaine seeking in extinguished animals, suggesting a potential “anti-relapse” function of this projection [49–51]. How does the connectivity change in these glutamate inputs to NAcSh ensembles during abstinence? The synaptic modulation of NAcSh ensembles from these glutamatergic projections induced by withdrawal from cocaine deserves further investigation.

### Spontaneous ensemble excitability is increased during prolonged withdrawal, which enhances craving for cocaine

Studies show that withdrawal of cocaine changes neuronal activity [52–54]. In line with these results, we found that greater activation of NAcSh cocaine-ensembles was induced by drug-seeking tests after 30-day withdrawal, and the neuronal activation of NAcSh cocaine-ensembles was correlated with the degree of cocaine craving. Our data also suggest that the membrane excitability in NAcSh cocaine-ensembles that encode cocaine memory is dynamically enhanced by withdrawal. The NAcSh ensembles showed a decrease of rheobase and an increase of RMP and input resistance after abstinence. The active and passive membrane properties of neurons, such as NAc MSNs, are primarily regulated by the inward-rectifying potassium currents [55–57]. It has been demonstrated that Kir_2.1_ channels are the major target for neuromodulation in the NAc [31]. Intrinsic excitability in D1-MSNs is enhanced after footshock stress through decreased IRK currents [19]. In this study, we found that Kir_2.1_ expression and currents were decreased selectively in NAcSh cocaine-ensembles after 30-day withdrawal, and selective Kir_2.1_ expression in NAcSh cocaine-ensembles reversed membrane properties and suppressed cocaine-seeking behavior after prolonged abstinence. Our study provides a pathological framework wherein withdrawal increases membrane excitability in NAcSh cocaine-ensembles through decreased IRK currents, which leads to an increased craving for cocaine. We prove that IRK-mediated enhancement of membrane excitability is critical for incubation of cocaine craving. It is reported that Kir channel activity is strongly dependent on intracellular regulators, such as phospholipids, kinase, ions, and guanosine-triphosphate binding proteins (G-proteins). With the TRAP data, we found that *Pip4p1* (phosphatidylinositol-4,5-bisphosphate 4-phosphatase 1), *Pip4k2b* (phosphatidylinositol-5-phosphate 4-kinase 2b), and *Pip4k2c* (phosphatidylinositol-5-phosphate 4-kinase 2c) showed significant downregulation (fold change: 0.58, 0.60, 0.64, respectively) after 30-day withdrawal. These genes are closely related to phosphatidylinositol-4,5-bisphosphate (PIP2). Kir channels are gated by the interaction of their cytoplasmic regions with membrane-bound PIP2 [58]. The prolonged withdrawal might impair functions of Kir2.1 channel through the downregulation of PIP2 signaling pathway. The importance of IRK channels as therapeutic targets in mood disorders is rapidly emerging [59, 60]. Thus, our results suggest that Kir_2.1_ is the potential target for clinical treatment of addiction, and activation of Kir_2.1_ decreases craving for cocaine presumably by decreasing excessive excitability of the ensembles.

Previous studies from noncontingent cocaine injection show that intrinsic membrane excitability of NAcSh neurons is decreased after a short-term abstinence that persists for at least 2 weeks [15, 16, 61]. Notably, cocaine SA and early withdrawal trigger the first round of synapse-membrane homeostatic crosstalk (SMHC) to decrease the membrane excitability of NAcSh MSNs, which initiates the second round of SMHC, resulting in further strengthening of NAc excitatory synapses and decreases in membrane excitability after long-term withdrawal from cocaine. Thus, the membrane excitability of NAcSh MSN is persistently decreased after prolonged withdrawal from cocaine-SA [4]. However, repeated cocaine injection induces increased firing capacity in the NAccore during early abstinence that declines to basal levels within 2 weeks [61]. These studies suggest that, when compared to saline treatment and randomly sampled, MSNs in the NAcSh, but not the NAccore, exhibit decreased membrane excitability after withdrawal of cocaine. In this study, we performed electrophysiological recording randomly on the eGFP^−^ cells (non-ensembles) around the eGFP^+^ cells (ensembles) in both saline and cocaine-SA groups. Our results showed that, when compared with saline-SA, the AP frequency and input resistance were decreased, and rheobase was increased in the cocaine non-ensembles in the NAcSh after 1-day withdrawal (data not shown), suggesting a decrease of membrane excitability in NAcSh MSN after cocaine-SA training. Our study also showed an increase of rheobase in cocaine non-ensembles in the NAcSh after 30-day withdrawal from cocaine-SA (data not shown), suggesting a persistent decrease of intrinsic membrane excitability might be induced in cocaine non-ensembles by prolonged withdrawal. Our results of cocaine non-ensembles in the NAcSh are consistent with the previous studies. Importantly, our results show that the membrane excitability is progressively increased in cocaine ensembles in the NAcSh compared to cocaine non-ensembles, indicating the complexity in NAcSh MSN populations and their differential contributions to incubated cocaine seeking after drug withdrawal.

In conclusion, we provide evidence that cocaine-ensembles in the NAcSh play a critical role in incubation of cocaine craving, demonstrating a correlation between ensemble activity and incubation of cocaine craving. Our data show that prolonged withdrawal decreases the translational activity of *Kcnj2* and downregulates Kir_2.1_ currents in NAcSh cocaine-ensembles. Our results provide translational potential that the downregulation of NAcSh ensemble activity could be useful for the treatment of cocaine addicts.

## Supplementary information

Supplementary Information

Supplementary Figure 1

Supplementary Figure 2

Supplementary Figure 3

Supplementary Figure 4

Supplementary Figure 5

Supplementary Figure 6

Supplementary Figure 7

Supplementary Figure 8

Supplementary Figure 9

## Data Availability

TRAP RNA sequencing data have been deposited in the Gene Expression Omnibus under accession number PRJNA782428.
