# Supplementary material for: Persistent increase of accumbens cocaine ensemble excitability induced by IRK downregulation after withdrawal mediates the incubation of cocaine craving
Source: Mol Psychiatry. 2022 Dec 8;28(1):448–62. doi: 10.1038/s41380-022-01884-1 (PMC9812793; doi:10.1038/s41380-022-01884-1)
Supplement: Supplementary file 1 — Supplementary Information [file 41380_2022_1884_MOESM1_ESM.docx]

**Supplementary Information**

**Persistent increase of accumbens cocaine ensemble excitability induced by IRK downregulation after withdrawal mediates the incubation of cocaine craving**

Guanhong He, Ziqing Huai, Changyou Jiang, Bing Huang, Zhen Tian, Qiumin Le, Guangyuan Fan, Haibo Li, Feifei Wang, Lan Ma, Xing Liu

The file includes:

Supplementary materials and methods

Supplementary Figs. 1 to 9

Supplementary Tables. 1 to 3

**Supplementary Materials and Methods**

**Animal**

*Fos-tTA* mice (stock #018306), *Arc^CreER^* mice (stock #021881) and *R26^AI14^* (*AI14*) mice (stock #007914) were purchased from The Jackson Laboratory. *Drd1-tdTomato* mice (stock #030512-UNC) and *Drd2-EGFP* mice (stock #000230-UNC) were purchased from the Mutant Mouse Resource and Research Center (MMRRC). *Arc^CreER^* mice crossed with *AI14* mice (*ArcTRAP;AI14*) were used for neuronal ensemble labeling in this study. The standard PCR protocols provided by The Jackson Laboratory were performed for genotyping of transgenic mice (Supplementary Table 3). C57/BL6J mice were purchased from the Shanghai Laboratory Animal Center, CAS. Adult male mice (P40-P90) were used for all experiments. Mice were housed under standard conditions of a reversed 12 h light/dark cycle with access to food and water ad libitum. The behavioral experiments were carried out during the light cycle. Mice were randomly assigned to experimental and control groups. All animal treatments were strictly in accordance with the National Institutes of Health Guide for the Care and Use of Laboratory Animals and were approved by Animal Care and Use Committee of Shanghai Medical College of Fudan University.

**Reagents**

Cocaine-hydrochloride (Qinghai Pharmaceutical Firm, China) and Clozapine-N-oxide (CNO, #C0832, Sigma-Aldrich, USA) were dissolved in saline. Tamoxifen (TAM, #T5648, Sigma-Aldrich, USA) was dissolved at 25 mg/ml in corn oil (Acros, Belgium).

**Preparation of Adeno-Associated Viruses (AAVs)**

The *pAAV-TRE-mCherry* and *AAV-c-Fos-tTA* constructs were the gifts from S. Tonegawa (RIKEN-MIT Center). *AAV_9_-TRE3g-Cre*, *AAV_9_-EF1α-DIO-Kir_2.1_-eGFP, AAV_9_-EF1α-DIO-DM-Kir_2.1_-tdTomato, AAV_9_-EF1α-FLEX-GCaMP7b* were packaged by BrainVTA Co., Ltd. *AAV_8_-c-Fos-tTA*, *AAV_9_-hSyn-DIO-hM4D-mCherry*, and *AAV_9_-hSyn-DIO-hM3D-mCherry* were packaged by Neuron Biotech Co., Ltd. *AAV_9_-TRE-tight-mCherry*, *AAV_9_-TRE-tight-eGFP, AAV_9_-EF1α-DIO-tdTomato*, and *AAV_9_-EF1α-FLEX-NBL10* were packaged by Taitool Biological Co., Ltd. AAV preparations with a titer exceeding 2 × 10^12^ vector genome per ml were used.

**Stereotaxic Surgery**

For viral microinjection, mice were anesthetized with isoflurane (3.5% for induction, 1.5-2% for maintenance), placed in a mouse stereotaxic instrument (Beijing Xingling Biotech Co., Ltd.), and injected with 100-200 nl AAVs with a Nano Microinjector (Beijing Xingling Biotech Co., Ltd.) aimed at NAcSh (AP: + 1.60 mm; ML: ± 0.50 mm; DV: – 4.80 mm) at the rate of 0.05 μl/min. The injectors were left in place for 10 minutes before withdrawal. 400 μm diameter optic fiber cannula (0.50 numerical aperture) was implanted unilaterally above NAcSh, the coordinates were: AP + 1.60 mm; ML ± 1.30 mm; DV – 4.40 mm (with an angle of 10° from the middle to the lateral). Animals were put on a warm heating pad for recovery.

**Behavioral Experiments**

***Jugular vein catheter.*** Food self-administration was conducted first to nose poke for food pellets (14 mg, catalog #F05684, Bio-serv, USA) under a fixed ratio 1 reinforcement schedule. Then the mice were anesthetized with isoflurane (3.5% induction, 1.5-2% maintenance, RWD, China) and implanted with an indwelling jugular catheter (#781-43-2202, Braintree Scientific Inc., USA). The mice were singly housed after surgery and were allowed to recover for 7 days before the start of cocaine-SA training. Catheters were flushed daily with a heparin solution (30 IU, #H3149, Sigma-Aldrich, USA), containing 0.33 mg/ml gentamicin (#A506614, Sangon Biotech, Shanghai, China) during the recovery period. Mice that failed in food-SA training were excluded.

***Cocaine-SA training*.** Cocaine self-administration was carried out in mouse operant chamber (ENV-307W-CT, Med Associates, USA) situated in an Expand PVC sound-attenuating cubicle (ENV-022V). Two nose poke holes were on the same side of one wall of the chamber with a cue-light located above each nose poke and a house light was present in each chamber. During the acquisition sessions, the stainless-steel tubing of the catheter device was connected through a 25-gauge swivel (PHM-124B) and a variable speed syringe pump (PHM-100VS-2c). Data were captured by a PC running MED-PC IV.

The mice were trained to self-administer cocaine for 10 sessions (1 session/day). Each session lasted for 4 h (0.5 mg/kg/infusion) or 6 h (1.5 mg/kg/infusion). Each active nose poke resulted in a cocaine infusion (0.5 or 1.5 mg/kg/infusion, dissolved in 0.9% saline and delivered at 7.97 ul/s) and a tone-light cue (10 s, 2900 Hz tone with cue light above the nose poke while the house light turned off). Active nose poke triggered a timeout period of 40 s. Poking in the inactive hole had no consequences. The active nose pokes were randomly assigned for each mouse. To avoid overdose, a maximum of 100 infusions were allowed per session for the dose of 0.5 mg/kg/infusion, and a maximum of 50 infusions were allowed for the dose of 1.5 mg/kg/infusion.

***Withdrawal and drug-seeking test.*** We assessed incubation of cue-induced cocaine craving in drug-seeking tests conducted in the same mouse after the various time of withdrawal from cocaine self-administration. During withdrawal, mice were kept in their home cage. The drug-seeking test was performed for 30 min or 60 min, during which, active nose pokes resulted in contingent delivery of the tone-light cue, identical to the cocaine training procedure but without cocaine infusion. Pump noise was maintained.

***Neuronal ensemble labeling*.** In cocaine-SA model, to label neuronal ensembles with tdTomato activated by cocaine self-administration, *ArcTRAP;AI14* mice were injected with Tamoxifen (125 mg/kg, i.p.) on day 6 after 5-day SA training and rested for 24 h before labeling. On day 7, cocaine-SA training was carried out (cocaine-ensemble labeling). The *Fos-tTA* transgene was also introduced in this study. The *Fos-tTA* mice were raised on food containing doxycycline (Dox, 40 mg/kg, # HY-N0565B, MedChemExpress, USA). To label NAcSh ensembles with hM4D, hM3D, mCherry, eGFP, NBL10, Kir_2.1_, or DM-Kir_2.1_, regular food without Dox was provided on day 5 after cocaine-SA training and cocaine-SA training (cocaine-ensemble labeling) or a novel context exposure (control-ensemble labeling) was performed on day 7. Immediately after this performance, Dox diet (1 g/kg) and Dox water (200 mg/L) were provided to stop further labeling.

***Open field test (OFT).*** The open field tests were performed in a monitor system (43.2 cm length × 43.2 cm width × 30.5 cm height, MED Associates, USA). Each mouse was placed in the center of the open field and allowed free exploration for 30 min. A square of 50% of the total area was defined as the center zone. The total distance traveled and the time spent in the center zone were recorded.

***Elevated plus maze (EPM).*** The elevated plus maze tests were performed on the equipment consisting of four arms (34.5 cm length × 6.3 cm width × 19.5 cm height). The elevated plus maze was placed 75 cm above the ground in a dimmed room. Two arms had 20 cm dark walls (closed arm) and the other two had 0.8 cm high ledges (open arm). Mice were placed in the center and allowed free exploration for 6 min. The behaviors were tracked and analyzed by Ethovision XT software (Noldus, Wageningen, Netherlands).

**Immunohistochemistry (IHC)**

Mice were anesthetized with isoflurane (3.5% induction, 1.5-2% maintenance, RWD, China) and transcardially perfused with 0.9% saline followed by 4.0% paraformaldehyde in 0.1 M Na_2_HPO_4_/NaH_2_PO_4_ buffer (pH = 7.4). The whole brain was quickly removed, processed for post-fixing in 4.0% paraformaldehyde for 4 h, and dehydrated with 30% sucrose for 3 days. Brain slices were sectioned into 30-μm frozen coronal slice by a CM3050 S Cyostat (Leica, Germany). Following blocking in 5.0% normal goat serum (Jackson ImmunoResearch, USA) in PBS (in mM: 10 Phosphate buffer, 137 NaCl, 2.7 KCl, 0.3% TritonX-100, pH 7.4), floating sections were incubated in the primary antibodies to c-Fos (Rabbit, 1:1000, #226003, Synaptic Systems, Germany)^1^, and Kir_2.1_ (Rabbit, 1:200, #APC026, Alomone Labs, Israel)^2^ at 4°C overnight. After rinsing in PBS, sections were incubated in fluorescence secondary antibody Cy3 or FITC IgG (mouse or rabbit, 1:50000, Jackson ImmunoResearch) at room temperature for 1 h. The sections were then washed and visualized under a Nikon A1 laser confocal fluorescence microscope (Nikon, Japan). Data were analyzed blindly to the group using Image-Pro Plus 6.0 and ImageJ (Fiji).

**Single-molecule RNA Fluorescence in Situ Hybridization (smFISH)**

Mice were anesthetized and transcardially perfused with 0.9% saline followed by 4.0% paraformaldehyde in 0.1 M Na_2_HPO_4_/NaH_2_PO_4_ buffer (pH = 7.4). After post-fixation and dehydration, 10-μm thick frozen brain slices were cut coronally and mounted onto Superfrost Plus microscope slides (Fisher Scientific, USA). FISH was performed following the RNAscope procedures (Advanced Cell Diagnostics, ACD, USA). Sections were incubated with probes against mouse *Drd1* and *Drd2* (*Drd1*, accession No: NM_010076.3, target region 521-1524; *Drd2*, accession No: NM_010077.2, target region 69-1175) for 2 h at 40℃. After hybridization, we used RNAscope® Multiplex Fluorescent Detection Kit v2 (#323110, ACD) to amplify the signal. Images were acquired and analyzed blindedly to the group allocation.

**Fiber Photometry**

An optical fiber with an outer diameter of 400 μm and 0.50 numerical aperture (Inper Tech, Hangzhou, Zhejiang, China) was implanted unilaterally into the NAcSh of mice and the fluorescence signals were recorded using a Fiber Photometry system equipped with 470- and 410-nm excitation lasers (Inper Tech). The laser power was adjusted at the tip of optical fiber to the low level of 10-15 μW at 410 nm and 25-30 μW at 470 nm. For comparing Ca^2+^ event frequency between WD1 and WD30 sessions, each animal was tested 1 h for 1 trial after 1-day and 30-day withdrawal in a MED-PC operant chamber, respectively, and signals were recorded for the entire duration. The signals were analyzed as follows: raw signals were adjusted to a flat baseline after baseline correction and motion correction using a script provided by Inper Tech; the baseline-adjusted signals were transformed to *ΔF/F* by dividing it by the mean of its raw signals. And then a value of 4 times the mean absolute deviation (4*MAD) of*ΔF/F* of the WD1 session was used as the threshold for detecting events for both WD1 and WD30 sessions, with a value of 95% of 4*MAD to re-arm event detection^3^. For the signals analysis relative to each active nosepoke, the 470 and 410 nm signals were independently processed and normalized to baseline signals to determine *ΔF/F, ΔF/F* = (*F*-*F_0_*) and *F_0_* is the mean value of the integral of the pre-stimulus signal (10 s). GCaMP signals were analyzed with MATLAB R2019b (MathWorks) and plotted with MATLAB as previously reported^4^. The mice with off-target fiber tip were excluded from the analysis.

**Ex vivo Electrophysiology Recording**

***Brain tissue preparation*.** Coronal slices (300 μm thick) containing the NAcSh were prepared as previously described^4, 5^. Mice were deeply anesthetized using isoflurane (3.5%) without test or 5 min after cue-induced drug-seeking test, and then perfused transcardially with 10 ml ice-cold and oxygenated (95% O_2_, 5% CO_2_) NMDG-based cutting solution containing the following (in mM): 92 N-methyl-D-glucamine diatrizoate, 93 HCl, 2.5 KCl, 1.25 NaH_2_PO_4_, 30 NaHCO_3_, 20 HEPES buffer, 25 glucose, 5 sodium ascorbate, 2 thiourea, 3 sodium pyruvate, 10 MgCl_2_, and 0.5 CaCl_2_ (pH = 7.3-7.4, 300-310 mOsm). The brains were rapidly removed and placed in ice-cold and oxygenated cutting solution. The coronal slices (300 μm) containing the NAcSh were prepared using a semiautomatic vibrating blade microtome (HM650V, Thermo) and then transferred to an incubation chamber at 32 °C with the oxygenated cutting solution for 10 min. After the initial recovery period, the slices were then kept at room temperature in modified artificial cerebrospinal fluid (ACSF) that contained (in mM): 94 NaCl, 2.5 KCl, 1.2 NaH_2_PO_4_, 30 NaHCO_3_, 20 HEPES, 25 glucose, 5 sodium ascorbate, 2 thiourea, 3 sodium pyruvate, 1.25 MgCl_2_ and 2.5 CaCl_2_ (pH = 7.3–7.4, 300–310 mOsm). For whole-cell patch-clamp recording, the slices were transferred to the recording chamber and perfused with 32 °C oxygenated recording ACSF containing (in mM): 124 NaCl, 2.5 KCl, 1.2 NaH_2_PO_4_, 24 NaHCO_3_, 5 HEPES, 12.5 glucose, 1.2 MgCl_2_ and 2.4 CaCl_2_ (pH = 7.3–7.4, 300–310 mOsm) at a rate of 3 ml/min. Slices were recorded within 6 h after preparation. Individual neurons were identified under a BX51WI microscope (Olympus, Tokyo, Japan) equipped with Rolera Bolt CCD camera (QImaging, Surrey, BC, Canada) with an EPC-10 amplifier and patchmaster software (HEKA Elektronik, Lambrecht/Pfalz, Germany). The pipette resistance was in the range of 8-10 MΩ. Current clamp recordings were filtered at 2.9 kHz and sampled at 5 kHz. The data were analyzed with Clampfit 10.3.

***Action protentional.*** K^+^-based intracellular solution (in mM) (130 K-gluconate, 6 KCl, 2 MgCl_2_, 10 HEPES, 2.5 ATP-Mg, 0.5 GTP-Na_2_, 10 creatine phosphate, 0.6 EGTA, 0.5% biocytin, pH = 7.25, 290 mOsm) was used for AP recording^6^. A current-step protocol (from -40 to +200 pA, with a 20-pA increment) lasting 1 s was run and repeated. After the recording of a particular cell, the number of evoked AP was compared across all runs; cells with a run-up or run-down >15% were excluded. After achieving whole cell configuration, resting membrane potential (RMP) was recorded without current injection. Rheobase was measured by injecting a variable positive current step (2 pA increment from the beginning of 0 pA) lasting 800 ms until the cell discharged a single AP^7, 8^. Membrane input resistance was measured by injecting a negative 100 pA current step lasting 1s while holding the membrane potential to -80 mV. The after-hyperpolarization potential (AHP) was sampled after the first AP spike, usually elicited by the rheobase current step. AP threshold was measured at the point of inflection when the slope exceeds 10 mV/ms. Fast afterhyperpolarization (fAHP) was identified as the peak amplitude of the current 3-4 ms after AP threshold. Medium AHP (mAHP) was measured as the peak amplitude of the current 15-100 ms after AP threshold. AP amplitude or peak was measured as the voltage change from threshold to AP peak. AP half-width was measured as the latency from threshold to AP peak. AP latency is the time from the onset of rheobase current step to AP threshold^9^.

***Electrophysiology recording of ensembles with CNO stimulation on hM3Dq or hM4Di******.*** To verify the effects of CNO’s stimulation on *hM3Dq* or *hM4Di*, NAcSh cocaine-ensembles were labeled with *hM3Dq-mCherry* or *hM4Di-mCherry* during cocaine-SA training and the ex-vivo recording were performed. Rheobase, input resistance, and action potential firing were recorded before and after CNO (10 μM) bath.

***Inward rectifying K current.*** I/V curve was recorded using a 10-mV voltage step from -150 mV to -40 mV while holding the membrane potential to -80 mV. IRK currents were measured by using the voltage clamp protocol in the presence of CsCl (1 mM, #C4036, Sigma-Aldrich, USA). As CsCl resistant currents were close to ACSF-conditioned leaky currents, the IRK current evoked with the voltage clamp protocol described above was used as a proxy for IRK currents without CsCl subtraction. Kir_2.1_ blocker ML133 HCL (50 μM)^10^ (#4549, Tocris, USA) was bath applied. Recordings with Rs >30 MΩ were excluded from statistical analysis.

**Membrane Kir_2.1_ Analysis**

Brain slices were acquired as previously described and membrane expression of Kir_2.1_ was analyzed as previously reported^10^. For Kir_2.1_ immunostaining, the slices were incubated with primary antibody in 0.5% Triton X-100 diluted in PBS at 4℃ overnight followed by 2 h incubation with secondary antibody at room temperature. Then slices were stained for DAPI and then cover-slipped with anti-quenching mounting medium (Thermo Fisher Scientific, USA). For image analysis, the ensembles (tdTomato^+^) and the non-ensembles (tdTomato^-^) adjacent to the ensembles (＜50 μm, on the same focal plane) from images were analyzed. In detail, the interest regions of cell membrane with DAPI signal extracted using Morphological Filters module and average membrane fluorescence intensity at 488 nm was measured in ImageJ (Fiji, NIH). Background fluorescence of a 20×20 μm^2^ area in the region void of Kir_2.1_ signal was measured and subtracted from the data. Two-way ANOVA analysis was employed and the experimenter was blind to the group.

**Ribosome-associated Transcripts**

***Purification of mRNA from neuronal ensembles with Ribo-tag.*** The purification procedure was modified and performed as previously reported^11^. *AAV-TRE-3g-Cre* and *AAV-FLEX-NBL10* were bilaterally injected into the NAcSh of *Fos-tTA* mice. Three weeks later, NAcSh cocaine- or saline-ensembles were labeled in cocaine-SA or saline-SA training. Mice were decapitated 1 day, 30 days, or 90 days after cocaine-SA or saline-SA training. The brain sections containing the NAcSh were quickly manually dissected and immediately homogenized in ice-cold supplemented hybridization buffer (in mM) (25 Tris [pH = 7.0], 25 Tris [pH = 8.0], 12 MgCl_2_, 100 KCl, 1% Triton X-100, 1 DTT (#646563-10X, Sigma-Aldrich, USA), 1×Protease inhibitors (#04693159001, Roche, Switzerland), 200 unit/ml RNase inhibitor (#N2112S, Promega, USA), 100 μg/ml cycloheximide (#14126, Cayman, USA), 1 mg/ml heparin (#H3149, Sigma-Aldrich). Homogenates were centrifuged for 10 min at 10,000 rpm, 4℃. RNA was extracted from 10% of cleared supernatant as input. The remaining supernatant lysate (output) was incubated with 3 μg Rabbit anti-HA (H6908, Sigma-Aldrich) for 4 hours and 100 μl Dynabeads Protein G (#10003D, Novex, USA) overnight at 4℃ with end-over-end rotation sequentially. Beads were collected on a magnetic rack, and washed three times with high-salt polysome wash buffer (in mM) (25 Tris [pH = 7.0], 25 Tris [pH = 8.0], 12 MgCl_2_, 300 KCl, 1% Triton X-100, 1 mM DTT, 100 μg/ml cycloheximide). Purified mRNA was eluted from the Dynabeads using SuPerfectRI Total RNA Isolation Reagent according to the manufacturer’s instructions with the inclusion of a DNase digestion step. Agilent RNA 6000 Pico Kit (5067-1513, Agilent, USA) and Agilent 2100 bioanalyzer were used to evaluate the quality and concentration of purified mRNA.

***Next-generation sequencing.*** mRNA was enriched using NEB Next Poly(A) mRNA Magnetic Isolation Module (NEB, E7490S, Ipswich, MA, USA). The library was prepared with VAHTS Total RNA-seq (H/M/R) Library Prep Kit for Illumina (NR603, Vazyme Biotech Co., Ltd, Jiangsu, China) and sequenced on a Novaseq (Illumina) by Genewiz (Jiangsu, China). Raw reads were quality-checked using FastQC and trimmed with Trimmomatic to remove adapter contamination and low-quality reads. The clean reads were aligned to mouse reference sequence (GRCm38/mm10) using HISAT2. Mapped reads for each transcript were counted using featureCounts and differential expression analysis was performed with DESeq2. Genes with more than twofold expression changes and significant differences (*p* < 0.05) were considered differentially expressed genes for further analysis. Clusterprofiler was used for signaling pathway enrichment and Cytoscape was used for network construction.

***Reverse transcription and qPCR.*** Reverse transcription was completed using the PrimeScript RT reagent Kit (#RR037A, Takara Biotechnology, Dalian, China). The cDNA was subjected to qPCR using SYBR Premix Ex Taq (#RR420A, Takara Biotechnology) and Eppendorf Mastercycler PCR System (Eppendorf, Germany). The primers were synthesized by Genewiz and listed in Supplementary Table 3.

**Statistical Analysis**

Data were analyzed with SPSS (IBM, Armonk, NY, USA) and plotted by Graphpad Prism. Our sample sizes were based on our previous research^6, 12^. Kolmogorov-Smirnov test was used for analyzing the cumulative distribution. Single-variable comparisons between groups were analyzed with two-tailed *student’s t-*test or one-way ANOVA. Two-variable comparisons between multiple groups were analyzed using two-way ANOVA, followed by *Bonferroni’s* post-hoc test. In detail, the results of behavioral test were analyzed by two-tailed paired *student’s t-*test or repeated measure (RM) two-way ANOVA, followed by *Bonferroni’s* post-hoc test with sessions as a within-subjects factor and CNO treatment or Kir_2.1_/DM-Kir_2.1_ expression as a between-subjects factor. The electrophysiological data were analyzed using two-tailed *Student’s t*-test, or two-way RM ANOVA followed by *Bonferroni’s* post hoc tests with current injection as a within-subjects factor and neuronal type as a between-subjects factor. Immunofluorescence data were analyzed by two-tailed *Student’s* t-test, Mann-Whitney U test, or one-way ANOVA, followed by *Bonferroni’s* post hoc tests. Data are presented as mean ± SEM.

**Data and Code Availability**

TRAP RNA sequencing data have been deposited in the Gene Expression Omnibus under accession number PRJNA782428.

**Reference**

1. Kim WB, Cho JH. Encoding of contextual fear memory in hippocampal-amygdala circuit. *Nat Commun.* 2020; **11:** 1382.

2. Murata Y, Yasaka T, Takano M, Ishihara K. Neuronal and glial expression of inward rectifier potassium channel subunits Kir2.x in rat dorsal root ganglion and spinal cord. *Neurosci Lett.* 2016; **617:** 59-65.

3. Pribiag H, Shin S, Wang EH, Sun F, Datta P, Okamoto A *et al.* Ventral pallidum DRD3 potentiates a pallido-habenular circuit driving accumbal dopamine release and cocaine seeking. *Neuron.* 2021; **109:** 2165-82 e10.

4. Jiang C, Yang X, He G, Wang F, Wang Z, Xu W *et al.* CRH(CeA-->VTA) inputs inhibit the positive ensembles to induce negative effect of opiate withdrawal. *Mol Psychiatry.* 2021; **26:** 6170-86.

5. Ting JT, Daigle TL, Chen Q, Feng G. Acute brain slice methods for adult and aging animals: application of targeted patch clamp analysis and optogenetics. *Methods Mol Biol.* 2014; **1183:** 221-42.

6. Jiang C, Wang X, Le Q, Liu P, Liu C, Wang Z *et al.* Morphine coordinates SST and PV interneurons in the prelimbic cortex to disinhibit pyramidal neurons and enhance reward. *Mol Psychiatry.* 2021; **26:** 1178-93.

7. Kim J, Park BH, Lee JH, Park SK, Kim JH. Cell type-specific alterations in the nucleus accumbens by repeated exposures to cocaine. *Biol Psychiatry.* 2011; **69:** 1026-34.

8. Gertler TS, Chan CS, Surmeier DJ. Dichotomous anatomical properties of adult striatal medium spiny neurons. *J Neurosci.* 2008; **28:** 10814-24.

9. Whitaker LR, Warren BL, Venniro M, Harte TC, McPherson KB, Beidel J *et al.* Bidirectional Modulation of Intrinsic Excitability in Rat Prelimbic Cortex Neuronal Ensembles and Non-Ensembles after Operant Learning. *J Neurosci.* 2017; **37:** 8845-56.

10. Pignatelli M, Ryan TJ, Roy DS, Lovett C, Smith LM, Muralidhar S *et al.* Engram Cell Excitability State Determines the Efficacy of Memory Retrieval. *Neuron.* 2019; **101:** 274-84 e5.

11. Sanz E, Yang L, Su T, Morris DR, McKnight GS, Amieux PS. Cell-type-specific isolation of ribosome-associated mRNA from complex tissues. *Proc Natl Acad Sci U S A.* 2009; **106:** 13939-44.

12. Zhou Y, Zhu H, Liu Z, Chen X, Su X, Ma C *et al.* A ventral CA1 to nucleus accumbens core engram circuit mediates conditioned place preference for cocaine. *Nat Neurosci.* 2019; **22:** 1986-99.

**Supplementary Figure Legends**

**Supplementary Fig. 1. Optimization of cocaine-SA incubation mouse models.**

**a-e** Cocaine-SA training was performed on days 1-5 and 7-11 at high dose (1.5 mg/kg/infusion, 6 h/day) or low dose (0.5 mg/kg/infusion, 4 h/day). Drug-seeking tests (1 h) were performed after withdrawal as indicated. **a** Experimental scheme. **b, d** Plots of active and inactive nose pokes during high (**b**) or low (**d**) dose of cocaine-SA training. **c, e** Plots of active nose pokes after withdrawal with high (**c**) or low (**e**) dose of cocaine-SA training. [**c**: n = 14, *F_session_* (2, 31) = 6.722, *p* = 0.002, RM One-way ANOVA, WD1 vs WD30, *p* = 0.020 within tests; **e**: n = 11, *t*_(10)_ = 1.688 , *p* = 0.122, Two-tailed paired *t*-test]. ^*^*p* < 0.05 vs WD1.

**Supplementary Fig. 2. Drug-seeking test induced cocaine-ensemble activation in the NAccore, mPFC, dCA1 after 30-day withdrawal.**

**a-d** Cocaine-SA training was performed on days 1-5 and 7-11 in *ArcTRAP;Ai14* mice. TAM (125 mg/kg) was injected on day 6 and the mice were subjected to SA training on day 7. The mice were sacrificed without drug-seeking tests after 1-day or 30-day withdrawal for c-Fos immunostaining. Locomotor activities were tested 1-day after Tamoxifen or vehicle injection. **a** Experimental scheme. **b** Representative images of tdTomato and c-Fos expression in the NAcSh without seeking tests. Red: tdTomato, Green: c-Fos. Scale bar: 100 μm. **c-d** Bar graph of c-Fos^+^ cell number/mm^2^ (**c**) and c-Fos^+^ tdTomato^+^/tdTomato^+^ ratio (%) (**d**). [WD1 n = 6, WD30 n = 3; c-Fos^+^ cell number: *t* _(7)_ = 1.054, *p* = 0.327; c-Fos^+^ tdTomato^+^/tdTomato^+^: *t* _(7)_ = 0.105, *p* = 0.920, Two-tailed *Student’s t*-test]. **e** Total distance in the open field and duration in the center arena in OFT test after Tamoxifen injection (125 mg/kg, i.p.) [Vehicle n = 10, Tamoxifen n = 10, total distance, *t* _(18)_ = 0.685, *p* = 0.502; center zone, *t* _(18)_ = 1.075, *p* = 0.297, Two-Tailed *Student’s t* test]. **f-o** Cocaine-SA training was performed on days 1-5 and 7-11 in *ArcTRAP;Ai14* mice. TAM (125 mg/kg) was injected on day 6 and the mice were subjected to SA training on day 7. The mice were sacrificed 60 min after drug-seeking tests with 1-day or 30-day withdrawal for c-Fos immunostaining. **f** Experimental scheme. **g-m** Representative images of tdTomato and c-Fos expression in the NAccore (**g**), PrL (**h**), IL (**i**), BLA (**j**), CeA (**k**), DG (**l**) and dCA1 (**m**). Red: tdTomato, Green: c-Fos. Scale bar: 100 μm. **n, o** Bar graph of c-Fos^+^ tdTomato^+^/tdTomato^+^ ratio (%) (**n**) and c-Fos^+^ tdTomato^+^/c-Fos^+^ ratio (%) (**o**). [WD1 n = 7, WD30 n = 10, c-Fos^+^ tdTomato^+^/tdTomato^+^ (%): NAccore, *U* = 13 , *p* = 0.033, Mann-Whitney U test; PrL, *t* _(15)_ = 2.497, *p* = 0.025; IL, *t* _(15)_ = 1.729, *p* = 0.104; BLA, *t* _(15)_ = 0.078, *p* = 0.939; CeA, *t* _(15)_ = 3.235, *p* = 0.006; DG, *t* _(15)_ = 0.698, *p* = 0.496; dCA1, *t* _(15)_ = 2.680, *p* = 0.017, Two-tailed *Student’s* *t*-test; c-Fos^+^ tdTomato^+^/c-Fos^+^ (%): NAccore, *t* _(15)_ = 2.428, *p* = 0.028; PrL, *t* _(15)_ = 1.358, *p* = 0.195; IL, *t* _(15)_ = 1.912, *p* = 0.075; CeA, *t* _(15)_ = 0.888, *p* = 0.389; DG, *t* _(15)_ = 0.858, *p* = 0.404; dCA1, *t* _(15)_ = 0.561, *p* = 0.583, Two-tailed *Student’s* *t*-test; BLA, *U* = 33, *p* = 0.887, Mann-Whitney U test]. **p* < 0.05 and ***p* < 0.01 vs WD1.

**Supplementary Fig. 3. Drug-seeking test induced activation of NAcSh cocaine-ensembles returns after 90- and 120-day withdrawal.**

**a-f** Cocaine-SA training was performed on days 1-5 and 7-11 (1.5 mg/kg/infusion, 6 h/day) in *ArcTRAP;Ai14* mice. TAM (125 mg/kg, i.p.) was injected on day 6 and the mice were subjected to SA training on day 7. The mice were sacrificed 60 min after cue-induced drug-seeking test for c-Fos immunostaining after 1-day and 30-day, 90-day and 120-day withdrawal, respectively. **a** Experimental scheme. **b** Representative images of tdTomato and c-Fos expression in the NAcSh of *ArcTRAP;Ai14* mouse. Red: tdTomato; Green: c-Fos. Scale bar: 100 μm. **c-f** Bar graph of tdTomato^+^ cell number/mm^2^ (**c**), c-Fos^+^ cell number/mm^2^ (**d**), c-Fos^+^ tdTomato^+^/tdTomato^+^ ratio (%) (**e**) or c-Fos^+^ tdTomato^+^/c-Fos^+^ ratio (**f**) in the NAcSh. [WD1 n = 7; WD30 n = 11; WD90 n = 5; WD120 n = 4, tdTomao^+^ cell number/mm^2^: *F_session_*(3.23) = 2.402, *p* = 0.094; c-Fos^+^ cell number/mm^2^: *F**_session_*(3.23) = 2.970, *p* = 0.053, WD1 vs WD30, *p* = 0.024 within tests; c-Fos^+^ tdTomato^+^/tdTomato^+^ (%): *F_session_* (3.23) = 5.992, *p* = 0.004, WD1 vs WD30, *p* = 0.002, WD30 vs WD120, *p* = 0.044 within tests; c-Fos^+^ tdTomato^+^/c-Fos^+^ (%): *F_session_* (3.23) = 5.646, *p* = 0.005, WD30 vs WD90, *p* = 0.028, WD30 vs WD120, *p* = 0.021 within tests, One-Way ANOVA]. ^*^*p* < 0.05, ^**^*p* < 0.01 vs indicated group.

**Supplementary Fig. 4. NAcSh cocaine-ensembles recruit mostly D1-MSNs.**

**a-c** *D1-tdTomato* mice were injected with *AAV-c-Fos-tTA* and *AAV-TRE-tight-eGFP,* and *D2-eGFP* mice were injected with *AAV-c-Fos-tTA* and *AAV-TRE-tight-mCherry* in the NAcSh. Cocaine-SA training was performed on days 1-5 and 7-11. For NAcSh cocaine-ensemble labeling, a regular diet without Dox was provided on days 6-7 to allow *c-fos*-driven expression of mCherry or eGFP, and Dox-containing diet (1 g/kg) was provided right after SA training on day 7. Mice were perfused on WD1. **a** Experimental scheme. **b** Representative image of eGFP^+^ cocaine-ensembles in *D1-tdTomato* mice and mCherry^+^ cocaine-ensembles in *D2-eGFP* mice. Red: tdTomato or mCherry, Green: eGFP, Scale bar: 100 μm. **c** Quantification of the percentage of D1^+^ or D2^+^ cocaine-ensembles [D1, n = 4 mice; D2, n = 5 mice]. **d-f** Cocaine-SA training was performed on days 1-5 and 7-11 (1.5 mg/kg/infusion, 6 h/day) in *ArcTRAP;Ai14* mice. TAM (125 mg/kg, i.p.) was injected on day 6 and the mice were subjected to SA training on day 7. Mice were sacrificed after 1-day withdrawal for smFISH. **d** Experimental scheme. **e** Representative image of tdTomato, *Drd1*, and *Drd2* expression in the NAcSh. Red: tdTomato, Green: *Drd1*, Grey: *Drd2*; Scale bar, left: 100 μm, right: 25 μm. **f** Quantification of the percentage of *Drd1*^+^ or *Drd2*^+^ ensembles in tdTomato^+^ cells [n = 5 mice].

**Supplementary Fig. 5. The activity of NAcSh saline-ensembles does not change after prolonged withdrawal.**

**a-e** *AAV-TRE-3g-Cre and AAV-FLEX-GCaMP7b* were injected in the NAcSh of *Fos-tTA* mice fed on diet containing Dox (40 mg/kg) and subjected to saline-SA training, and the optic fiber was unilaterally implanted for photometry. Regular diet without Dox was provided on days 6-7 to allow *c-fos*-driven expression of GCaMP. Photometry recording was performed during drug-seeking tests after 1-day and 30-day withdrawal. **a** Experimental scheme. **b** Sample GCaMP7b photometry trace of cocaine-ensembles during drug-seeking tests. Circles mark above trace indicate threshold-detected events. **c** Relative event frequency (% Normalized to WD1) of saline-ensembles during drug-seeking tests [n = 13, *t* _(12)_ = 0.142, *p* = 0.889, Two-tailed paired *t*-test]. **d** Representative image for GCaMP7b expression and trace of fiber in the NAcSh after 1-day and 30-day withdrawal. Green: eGFP; Blue: DAPI, Scale bar: 200 μm. **e** Mean intensity of the GCaMP density in NAcSh after 1-day and 30-day withdrawal [WD1 n =8, WD30, n = 7, *t* _(13)_ = 0.112, *p* = 0.913, Two-tailed *Student’s t*-test].

**Supplementary Fig. 6. Chemogenetic activation and inhibition of NAcSh ensembles.**

**a-f** *AAV-TRE-3g-Cre* and *AAV-hSyn-DIO-hM4D-mCherry* were injected in the NAcSh of *Fos-tTA* mice. NAcSh cocaine-ensembles and control-ensembles were labeled during cocaine-SA training but paired with different contexts. Mice were perfused 1-day after cocaine-SA training for ensemble counting and 30-day withdrawal for ex-vivo recording. **a** Experimental scheme. **b, c** Representative image (**b**) and bar graph (**c**) of the number of cocaine-ensembles and control-ensembles. Red, mCherry. Scale bar: 100 μm. [Control-ensemble n = 14, Cocaine-ensemble n = 11, *t*_(23)_ = 0.147, *p* = 0.884, Two-tailed *Student’s t*-test]. **d** Representative trace and the number of action potential under 80-pA stimulation recorded from hM4D-expressing NAcSh ensembles before (baseline) and 15 min after CNO application (10 μM). [n=11, *t* _(10)_ = 2.664, *p* = 0.024, Two-Tailed *paired t*-test]. **e-f** Rheobase (**e**) and input resistance (**f**) recorded from hM4D-expressing NAcSh ensembles before (baseline) and 15 min after CNO application. [n=11, Rheobase: *t* _(10)_ = 6.374, *p* ＜ 0.001, Rm: *t* _(10)_ = 2.529, *p* = 0.030,Two-Tailed *paired t*-test]. **g-l** *AAV-TRE-3g-Cre* and *AAV-hSyn-DIO-hM3D-mCherry* were injected in the NAcSh of *Fos-tTA* mice fed on diet containing doxycycline (Dox, 40 mg/kg). Cocaine-SA training was performed and cocaine-ensembles were labeled. After cocaine-SA training, the mice were sacrificed 90 min after CNO injection for c-Fos immunostaining and days later for ex-vivo recording. **g** Experimental scheme. **h, i** Representative images (**h**) and bar graph (**i**) of c-Fos^+^ cell numbers/mm^2^ in the NAcSh. [Vehicle n = 4, CNO n = 6, *t* _(8)_ = 4.593, *p* = 0.002, Two-Tailed *Student’s t* test]. **j** Representative trace and the number of action potential under 80-pA stimulation recorded from hM3D-expressing NAcSh ensembles before (baseline) and 15 min after CNO application (10 μM). [n=13, *t* _(12)_ = 2.278, *p* = 0.042, Two-Tailed *paired t*-test]. **k, l** Rheobase (**k**) and input resistance (**l**) recorded from hM3D-expressing NAcSh ensembles before (baseline) and 15 min after CNO application. [n=13, Rheobase: *t* _(12)_ = 2.708, *p* = 0.019, Rm: *t* _(12)_ = 4.640, *p* = 0.001, Two-Tailed *paired t*-test]. ^*^*p* < 0.05, ^**^*p* < 0.01, ^***^*p* < 0.001 vs indicated group.

**Supplementary Fig. 7. The membrane excitability of NAcSh cocaine-ensembles is enhanced after prolonged withdrawal.**

**a-i** *AAV-TRE-tight-eGFP* was injected in the NAcSh of *Fos-tTA* mice fed on diet containing Dox (40 mg/kg). Regular diet without Dox was provided on days 6-7 and cocaine-ensembles were labeled by SA training on day 7. The ex vivo recordings were performed after tests on 1-day and 30-day withdrawal. **a** Experimental scheme. **b** Plots of active and inactive nose pokes during cocaine-SA training. **c, d** Representative AP traces and graphs of AP frequency at the indicated current steps in NAcSh cocaine non-ensembles and ensembles. [non-ensemble: WD1 n = 25 cells/9 mice, WD30 n = 34 cells/11 mice, *F*_session×current_ (12, 684) = 0.520, *p =* 0.903; ensemble: WD1 n = 42 cells/9 mice, WD30 n = 49 cells/11 mice, *F*_session×current_ (12, 1068) = 4.312, *p* < 0.001, RM Two-way ANOVA]. **e-g** Representative trace and graphs of the resting membrane potential (RMP) (**e**), rheobase (**f**), and membrane resistance (Rm) (**g**) of NAcSh cocaine non-ensembles and ensembles [RMP: WD1 non-ensemble n = 30 cells/7 mice, WD30 non-ensemble n = 30 cells/7 mice, WD1 ensemble n = 31 cells/7 mice, WD30 ensemble n = 35 cells/7 mice, *F*_cell type×session_ (1, 122) = 6.260, *p* = 0.014, WD1 ensemble vs WD30 ensemble, *p* = 0.030 within tests; rheobase: WD1 non-ensemble n =31 cells/7 mice, WD30 non-ensemble n = 30 cells/7 mice, WD1 ensemble n = 32 cells/7 mice, WD30 ensemble n = 35 cells/7 mice, *F*_cell type×session_ (1, 124) = 6.234, *p* = 0.014, WD1 ensemble vs WD30 ensemble, *p* = 0.043 within tests ; Rm: WD1 non-ensemble n = 31 cells/7 mice, WD30 non-ensemble n = 30 cells/7 mice, WD1 ensemble n = 32 cells/7 mice, WD30 ensemble n = 35 cells/7 mice, *F*_cell type×session_ (1, 124) = 8.108, *p* = 0.005, WD1 ensemble vs WD30 ensemble, *p* = 0.005 within tests, Two-way ANOVA]. **g, h** Distribution of the rheobase and Rm of NAcSh cocaine non-ensembles (**g**) and ensembles (**h**). [WD1 non-ensemble n = 31 cells/7 mice, WD30 non-ensemble n = 30 cells/7 mice, Rheobase, *t*_(59)_ = 0.884, *p* = 0.380, Two-tailed *Student’s t*-test; Rm, *t*_(59)_ = 1.683, *p* = 0.098, Two-tailed *Student’s t*-test; WD1 ensemble n = 32 cells/7 mice, WD30 ensemble n = 35 cells/7 mice, Rheobase, *t*_(65)_ = 2.622, *p* = 0.011, Two-tailed *Student’s t*-test; Rm, *U* = 371, *p* = 0.017, Mann-Whitney U test]. ^*^*p* < 0.05, ^**^*p* < 0.01, ^***^*p* < 0.001, ^###^*p* < 0.001 vs indicated group.

**Supplementary Fig. 8. The Kir_2.1_ expression decreases in NAcSh cocaine-ensembles, but not in non-ensembles after prolonged withdrawal.**

**a-c** *AAV-TRE-3g-Cre* and *AAV-FLEX-NBL10* were injected in the NAcSh of *Fos-tTA* mice fed on a diet containing Dox (40 mg/kg). Saline-SA/Cocaine-SA training was performed and saline/cocaine-ensembles were labeled on day 7. The mouse brain sections containing the NAcSh were dissected and collected after 1-day and 90-day withdrawal for ribosomal mRNA purification. **a** Experimental scheme. **b, c** Quantification of relative mRNA levels of IRK family members in NAcSh cocaine-ensembles, saline-ensembles and total RNA. [WD1 n = 8, WD90 n = 8]. **d-e** *AAV-TRE-3g-Cre* and *AAV-FLEX-NBL10* were injected in the NAcSh of *Fos-tTA* mice fed on a diet containing Dox (40 mg/kg). Cocaine-SA training was performed and cocaine-ensembles were labeled on day 7. The mouse brain sections containing the NAcSh were dissected and collected after 1-day and 30-day withdrawal for ribosomal mRNA purification. **d** Experimental scheme. **e** Quantification of relative mRNA levels of K_v_, K_Ca_, K2P channel family members in NAcSh cocaine-ensembles after 1-day and 30-day withdrawal. [WD1 n = 7, WD30 n = 7]. **f-h** Cocaine-SA training was performed on *ArcTRAP;AI14* mice and cocaine-ensembles were labeled. The mice were sacrificed for Kir_2.1_ immunostaining after 1-day, 30-day, and 90-day withdrawal. **f** Experimental scheme. **g** Representative images of Kir_2.1_ expression in NAcSh of *ArcTRAP;Ai14* mice. Red: tdTomato, Green: Kir2.1, Blue: DAPI. Scale bar: 50 μm. **h** Quantification and cumulative probability curve of Kir_2.1_ expression. [WD1 Non-ensemble n = 96 cells/5 mice, WD30 Non-ensemble n = 91 cells/6 mice, WD90 Non-ensemble n = 92 cells/ 5 mice; WD1 ensemble n = 96 cells/5 mice, WD30 ensemble n = 91 cells/6 mice, WD90 ensemble n = 91 cells / 5 mice, *F* _cell type×session_ (2,551) = 2.665, *p* = 0.072, Two-way ANOVA;WD1 ensemble vs WD30 ensemble, *p* = 0.003, WD30 ensemble vs WD90 ensemble, *p* < 0.001 within tests, Cumulative probability curve: WD1 Non-ensemble vs WD30 Non-ensemble, *p* = 0.364, WD1 ensemble vs WD30 ensemble, p＜0.001, *Kolmogorov-Smirnov* test]. **i-m** Representative trace (**i**) of voltage-clamp recordings and IV curves (**j-l**) from D1-MSN before and following addition of CsCl (1 mM) and CsCl subtracted currents (**l**). **m** Bar graph of inward current before and following addition of CsCl. [n =11, *t*_(10)_ = 7.911, *p* < 0.001, Two-tailed *paired t*-test]. ^**^*p* < 0.01, ^***^*p* < 0.001 vs indicated group.

**Supplementary Fig. 9. The *Kcnj2* expression in NAcSh cocaine-ensembles does not alter locomotor and anxiety.**

**a-c** *AAV-TRE-3g-Cre* and *AAV-DIO-Kir_2.1_-eGFP* were injected in the NAcSh of *Fos-tTA* mice fed on diet containing Dox (40 mg/kg). Cocaine-SA training was performed and cocaine-ensembles were labeled. After 30-day withdrawal, locomotion and anxiety levels of mice were tested in open field tests (OFT) and elevated plus maze tests (EPM). **a** Experimental scheme. **b** Total distance in the open field and duration in the center arena in OFT [eGFP n = 6, Kir_2.1_ n = 7, total distance, *t* _(11)_ = 0.259, *p* = 0.800; time in the center zone, *t* _(11)_ = 0.635, *p* = 0.538, Two-Tailed *Student’s t* test]. **c** Time spent in the open (left) and closed (right) arm in elevated plus maze test. [eGFP n = 6, Kir_2.1_ n = 7, open arm, *t* _(11)_ = 0.588, *p* = 0.568; closed arm, *U* = 17, *p* = 0.628, Mann-Whitney U test]. **d-f** *AAV-TRE-3g-Cre* and *AAV-DIO-DM-Kir_2.1_-tdTomato* were injected in the NAcSh of *Fos-tTA* mice fed on diet containing Dox (40 mg/kg). Cocaine-SA training was performed and cocaine-ensembles were labeled. After 30-day withdrawal, locomotion and anxiety levels of mice were tested in open field tests (OFT) and elevated plus maze tasks (EPM). **d** Experimental scheme. **e** Total distance in the open field and duration in the center arena in OFT test. [tdTomato n = 6, DM-Kir_2.1_ n = 5, total distance, *t* _(9)_ = 0.055, *p* = 0.957; center zone, *t* _(9)_ = 0.368, *p* = 0.721, Two-Tailed *Student’s t* test]. **f** Time spent in the open (left) and closed (right) arm in elevated plus maze test. [tdTomato n = 6, DM-Kir_2.1_ n = 5, open arm, *t* _(9)_ = 0.224, *p* = 0.828; closed arm, *t* _(11)_ = 0.186, *p* = 0.857, Two-Tailed *Student’s t* test].

| **Table 1. Membrane properties：Cocaine-ensembles and -non-ensembles before and after prolonged withdrawal** | | | | |
| --- | --- | --- | --- | --- |
| Treatment | WD1 Non-ens | WD30 Non-ens | WD1 Coc-ens | WD30 Coc-ens |
| Number of cells | n=30 | n=30 | n=31 | n=35 |
| AP threshold (mV) | -27.260 ± 1.472 | -29.480 ± 1.267 | -27.550 ± 1.308 | -27.700 ± 1.022 |
| fAHP (mV) | -10.700 ± 0.539 | -10.860 ± 0.466 | -11.930 ± 0.607 | -12.260 ± 0.565 |
| mAHP (mV) | -9.467 ± 0.479 | -9.986 ± 0.441 | -9.739 ± 0.554 | -9.043 ± 0.439 |
| AP peak (mV) | 76.900 ± 1.350 | 80.070 ± 1.421 | 76.350 ± 1.592 | 77.230 ± 1.354 |
| Half Width | 1.572 ± 0.069 | 1.472 ± 0.063 | 1.324 ± 0.070* | 1.243 ± 0.069* |
| lantency (ms) | 402.100 ± 12.650 | 390.300 ± 13.830 | 365.700 ± 20.510 | 337.100 ± 18.710* |

**Supplementary Tables**

**Supplementary Table 1.** AP properties of NAcSh cocaine-ensembles and -non-ensembles after 1-day and 30-day withdrawal.

**p* < 0.05，WD30 Coc-ens vs WD30 Non-ens or WD1 Coc-ens vs WD1 Non-ens

**Supplementary Table 2.** AP properties of NAcSh saline-ensembles and -non-ensembles after 1-day and 30-day withdrawal.

| **Table 2. Membrane properties：Saline-ensembles and -non-ensembles before and after prolonged withdrawal** | | | | |
| --- | --- | --- | --- | --- |
| Treatment | WD1 Non-ens | WD30 Non-ens | WD1 Sal-ens | WD30 Sal-ens |
| Number of cells | n=28 | n=33 | n=28 | n=32 |
| AP threshold (mV) | -32.580 ± 1.285 | -32.210 ± 1.304 | -32.260 ± 1.066 | -31.350 ± 1.775 |
| fAHP (mV) | -12.600 ± 0.613 | -11.650 ± 0.878 | \| -11.500 ± 0.725 \| \| --- \| | -12.710 ± 0.630 |
| mAHP (mV) | -11.500 ± 0.569 | -10.020 ± 0.700 | -9.956 ± 0.577 | -10.920 ± 0.457 |
| AP peak (mV) | 81.600 ± 1.278 | 82.100 ± 1.289 | 79.520 ± 1.789 | 77.960 ± 1.847 |
| Half Width | 1.771 ± 0.060 | 1.626 ± 0.064 | 1.645 ± 0.066 | 1.571 ± 0.104 |
| lantency (ms) | 373.200 ± 18.730 | 384.500 ± 17.500 | 370.300 ± 20.680 | 360.500 ± 20.000 |

**Supplementary Table 3.** Primers for PCR

| **Gene** | **Primer** | **5’-3’** |
| --- | --- | --- |
| *gapdh* | gapdh-F | TGGCCTTCCGTGTTCCTAC |
|  | gapdh-R | GAGTTGCTGTTGAAGTCGCA |
| *kcnj1* | kcnj1-F | ACCCCTACAGCATCGTATCAT |
|  | kcnj1-R | GTTGCACTGACCGTTCTTCTT |
| *kcnj2* | kcnj2-F | AACCGCTACAGCATCGTCTC |
|  | kcnj2-R | GTTGTCGGGTATGGACTTTACTC |
| *kcnj3* | kcnj3-F | GGGGACGATTACCAGGTAGTG |
|  | kcnj3-R | CGCTGCCGTTTCTTCTTGG |
| *kcnj4* | kcnj4-F | ATGCACGGACACAACCGAAA |
|  | kcnj4-R | CTGGGACTTGTTGCTCAGG |
| *kcnj5* | kcnj5-F | GCCGGTGATTCTAGGAATGCT |
|  | kcnj5-R | TCACTTAGGTAGCGGTAGGTTT |
| *kcnb1* | kcnb1-F | AGAAACACACAGCAATAGCGT |
|  | kcnb1-R | GTACTCCCGTGGAGACTCTTG |
| *kcnb2* | kcnb2-F | TGTGGACATTATCCGAAGCAAA |
|  | kcnb2-R | TCTCGTGAGTGTTACAGTCGC |
| *kcnma1* | kcnma1-F | CGACAAACAGAATGCAACAAGG |
|  | kcnma1-R | ACTCATGGGCTTGATTTGAATGT |
| *kcnmb1* | kcnmb1-F | CCTGGGAGTGGCAATGGTAG |
|  | kcnmb1-R | CAAAGGCATGGGTACTGGGG |
| *kcnc3* | kcnc3-F | GGGCTTCTGGGGCATAGAC |
|  | kcnc3-R | GTCCTGAAAACACAGACGCTT |
| *kcnn2* | kcnn2-F | GCATGGACTGTCCGAGCTT |
|  | kcnn2-R | TTGGTGCAGATTCCCGTTCTT |
| *kcnn3* | kcnn3-F | TGTTGCACTCTTCTCCCACG |
|  | kcnn3-R | GGTCATTGAGATTTAGCTGGCT |
| *kcnq1* | kcnq1-F | GAACAGGGTGGAAGACAAGGTGA |
|  | kcnq1-R | GACCTCCCTTCTGAGCTCCTT |
| *kcnq2* | kcnq2-F | AGCAAATCTGGACTCACCTTCA |
|  | kcnq2-R | AGGGAGGCTTGCTTCTTCTG |
| *kcnmb4* | kcnmb4-F | TAATCAGCATCAGAGACCAGAG |
|  | kcnmb4-R | ACCACGATGAGAACACCCAC |
| *kcnk1* | kcnk1-F | GTGCTCTCCACCACAGGCTAT |
|  | kcnk1-R | AAGAGGAGGGTGAACGGGAT |
| *kcnk2* | kcnk2-F | CTATACTGCAGGAGTGGCGG |
|  | kcnk2-R | CAAGCACGGTGGGTTTTGAG |
| *kcnk3* | kcnk3-F | CGATGAAGCGGCAGAATGTG |
|  | kcnk3-R | CCGCATGACCATAGCCGAT |
| *kcnk6* | kcnk6-F | CAAGGTGCTTGTCACAGCGTA |
|  | kcnk6-R | TCGGGCAGCAAGATGAGTTC |
| *kcnk10* | kcnk10-F | TCGTGGTCTACCTCGTCACT |
|  | kcnk10-R | AGCATCGAGTGCATGCTGAA |
| **Type of mouse** | **Primer** | **5’-3’** |
| *Arc^CreER^* | Common | CAGCATAAATAGCCGCTGGT |
|  | Wild type Reverse | CCGTCCAAGTTGTTCTCCAG |
|  | Mutant Reverse | CGACCGGTAATGCAGGC |
| *R26^Ai14/+^* (*Ai14*) | Wild type Forward | AAGGGAGCTGCAGTGGAGTA |
|  | Wild type Reverse | CCGAAAATCTGTGGGAAGTC |
|  | Mutant Reverse | GGCATTAAAGCAGCGTATCC |
|  | Mutant Forward | CTGTTCCTGTACGGCATGG |
| *Fos-tTA* | Forward | ACCTGGACATGCTGTGATAA |
|  | Reverse | TGCTCCCATTCATCAGTTCC |
