# Supplementary figures and images for: Persistent increase of accumbens cocaine ensemble excitability induced by IRK downregulation after withdrawal mediates the incubation of cocaine craving
Source: Mol Psychiatry. 2022 Dec 8;28(1):448–62. doi: 10.1038/s41380-022-01884-1 (PMC9812793; doi:10.1038/s41380-022-01884-1)

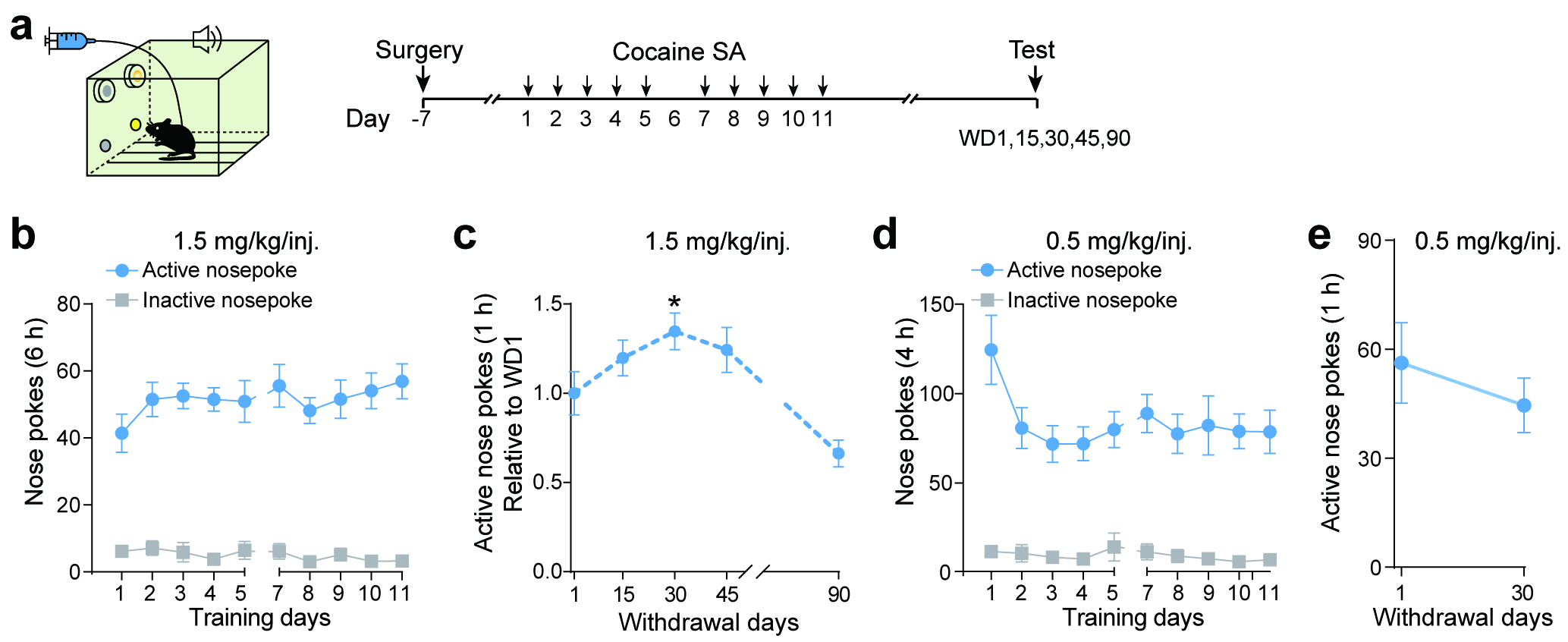

Supplement: Supplementary file 2 — Supplementary Figure 1 [file 41380_2022_1884_MOESM2_ESM.tif]

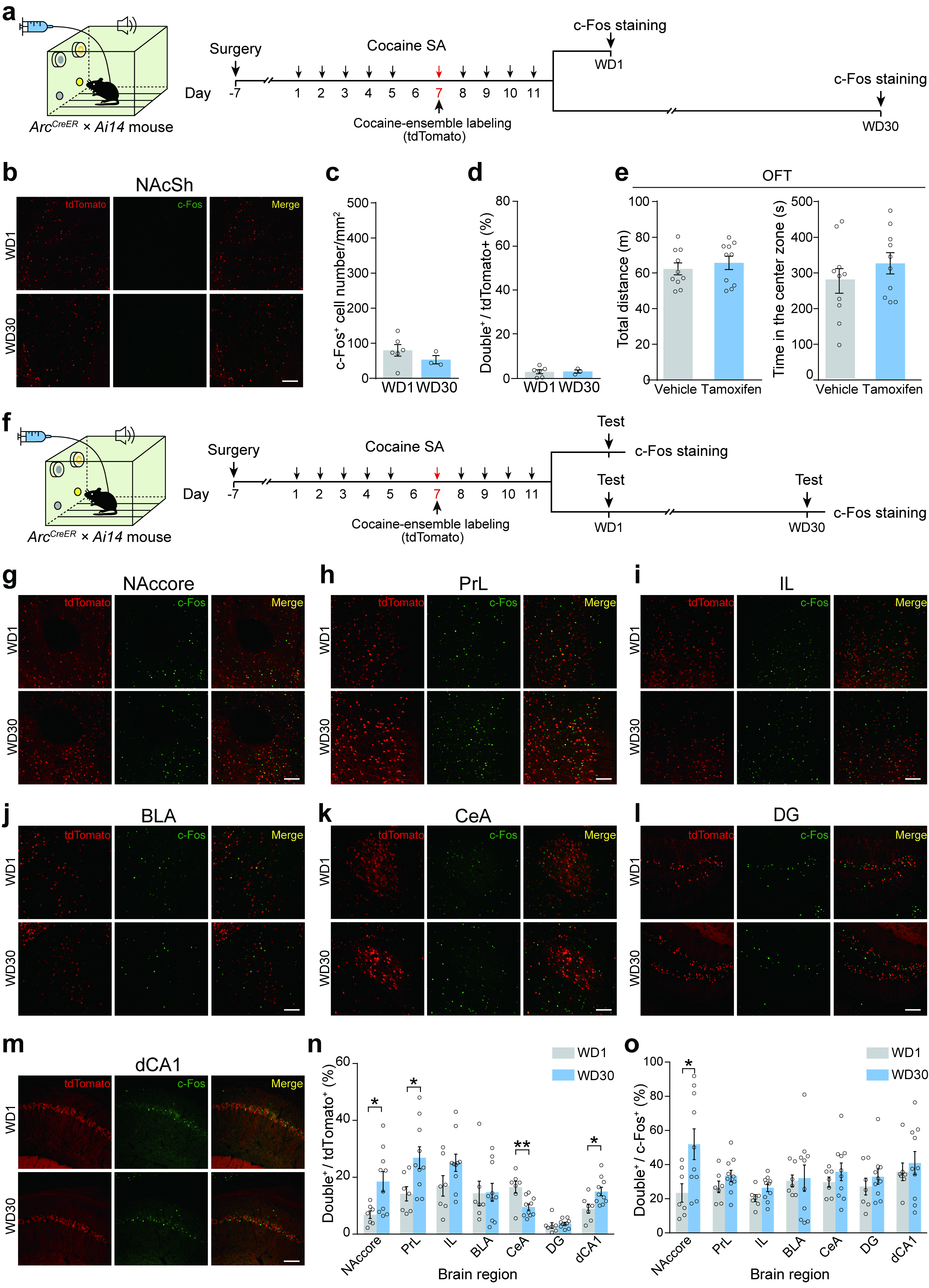

Supplement: Supplementary file 3 — Supplementary Figure 2 [file 41380_2022_1884_MOESM3_ESM.tif]

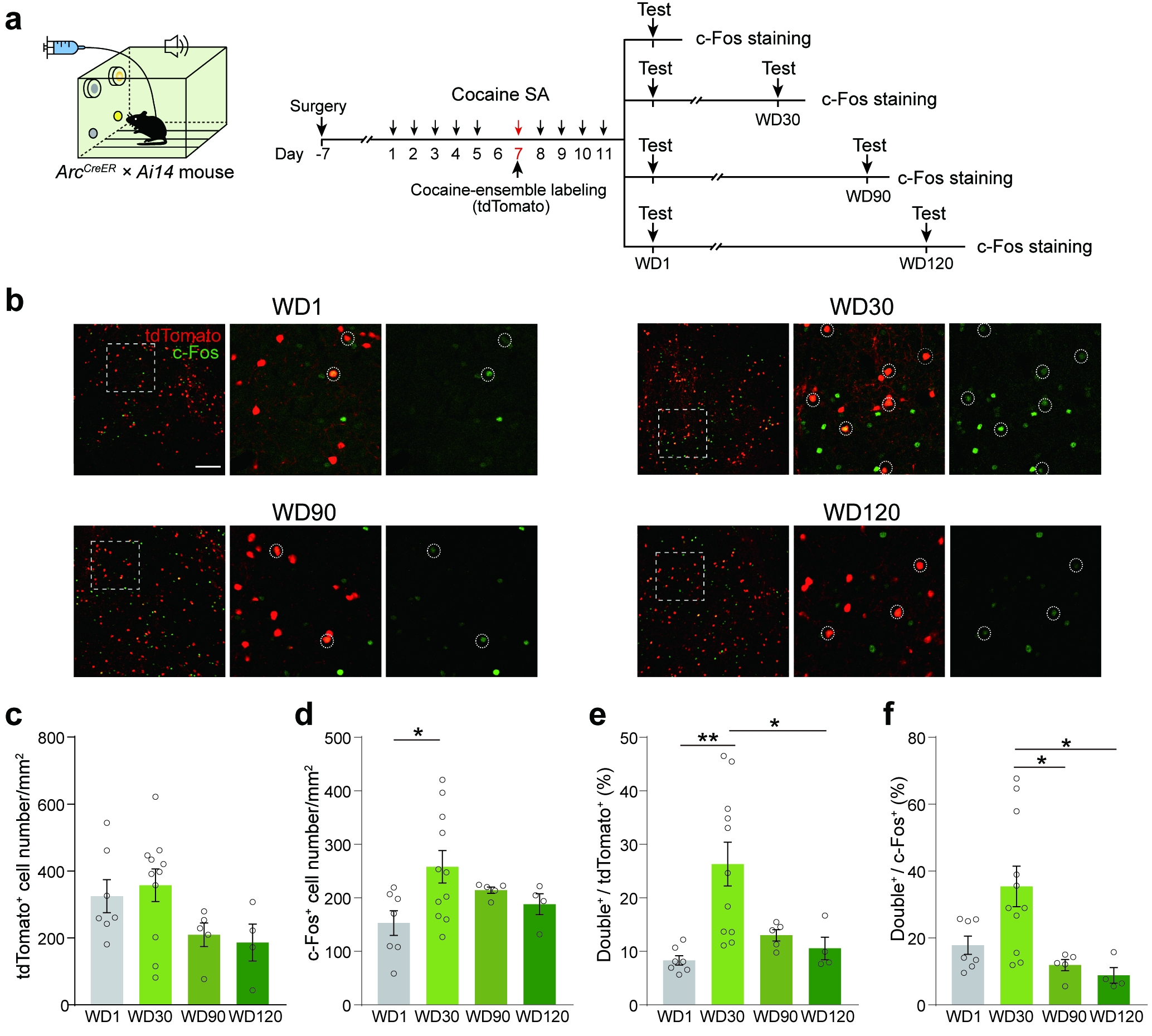

Supplement: Supplementary file 4 — Supplementary Figure 3 [file 41380_2022_1884_MOESM4_ESM.tif]

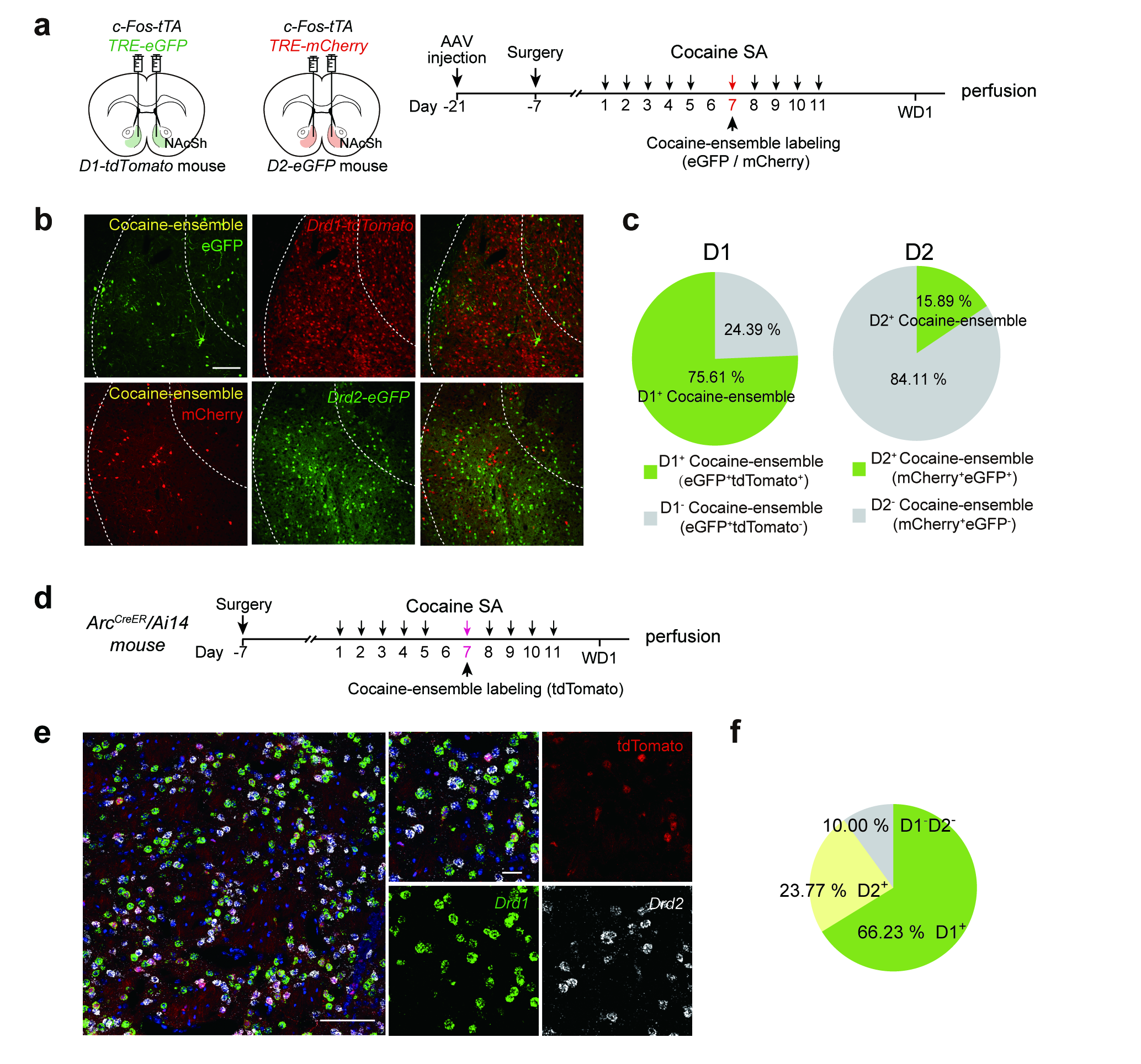

Supplement: Supplementary file 5 — Supplementary Figure 4 [file 41380_2022_1884_MOESM5_ESM.tif]

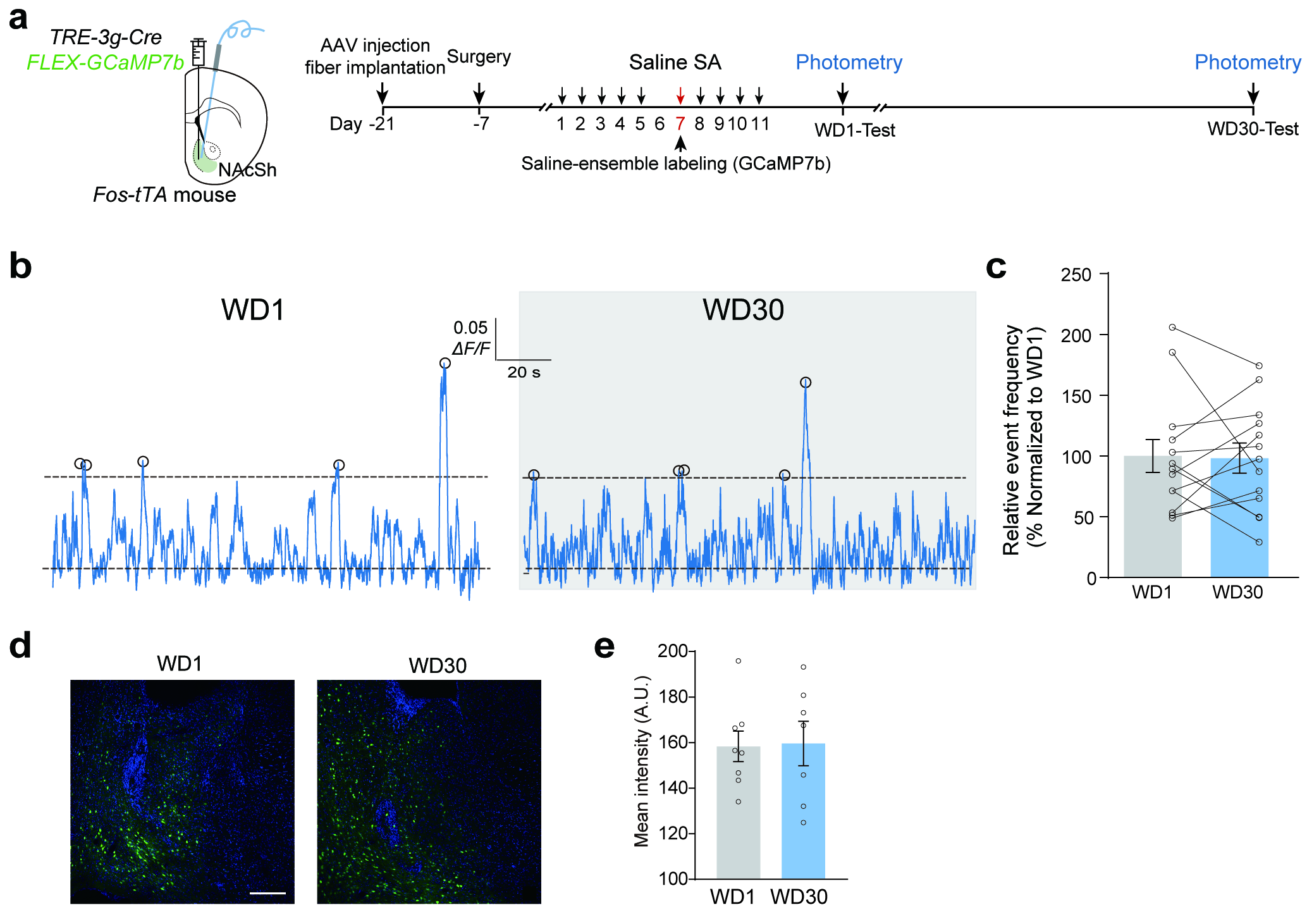

Supplement: Supplementary file 6 — Supplementary Figure 5 [file 41380_2022_1884_MOESM6_ESM.tif]

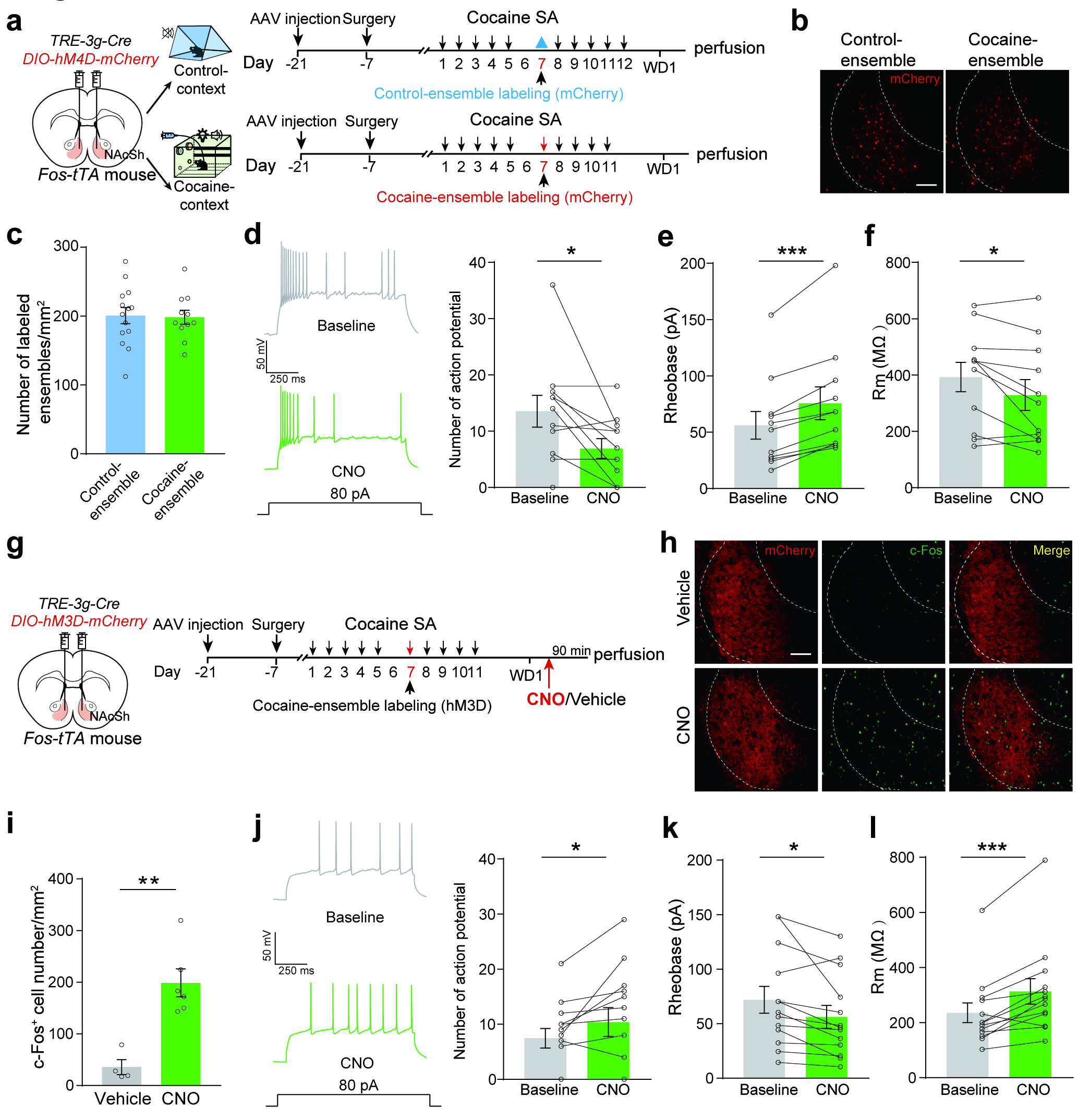

Supplement: Supplementary file 7 — Supplementary Figure 6 [file 41380_2022_1884_MOESM7_ESM.tif]

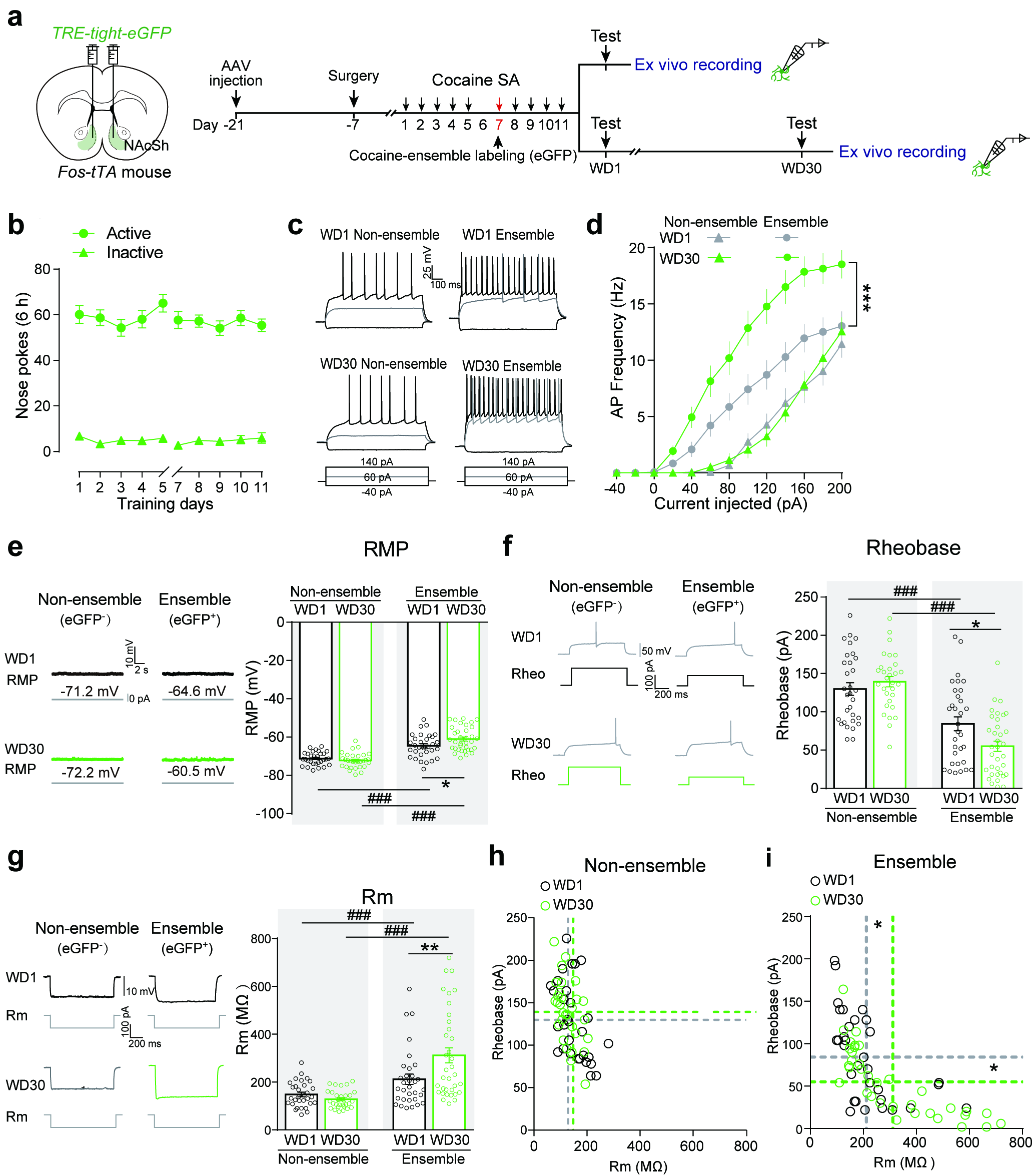

Supplement: Supplementary file 8 — Supplementary Figure 7 [file 41380_2022_1884_MOESM8_ESM.tif]

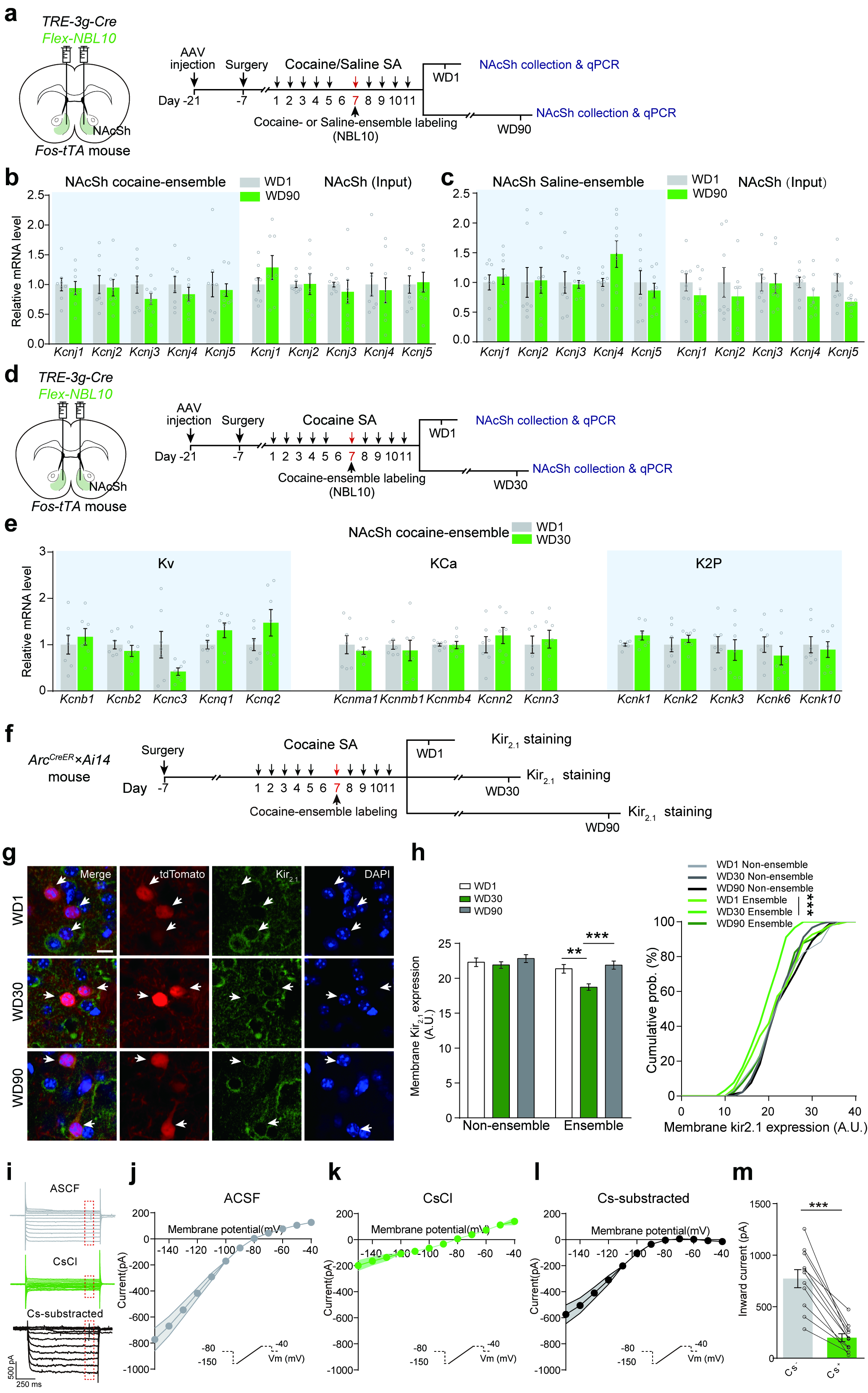

Supplement: Supplementary file 9 — Supplementary Figure 8 [file 41380_2022_1884_MOESM9_ESM.tif]

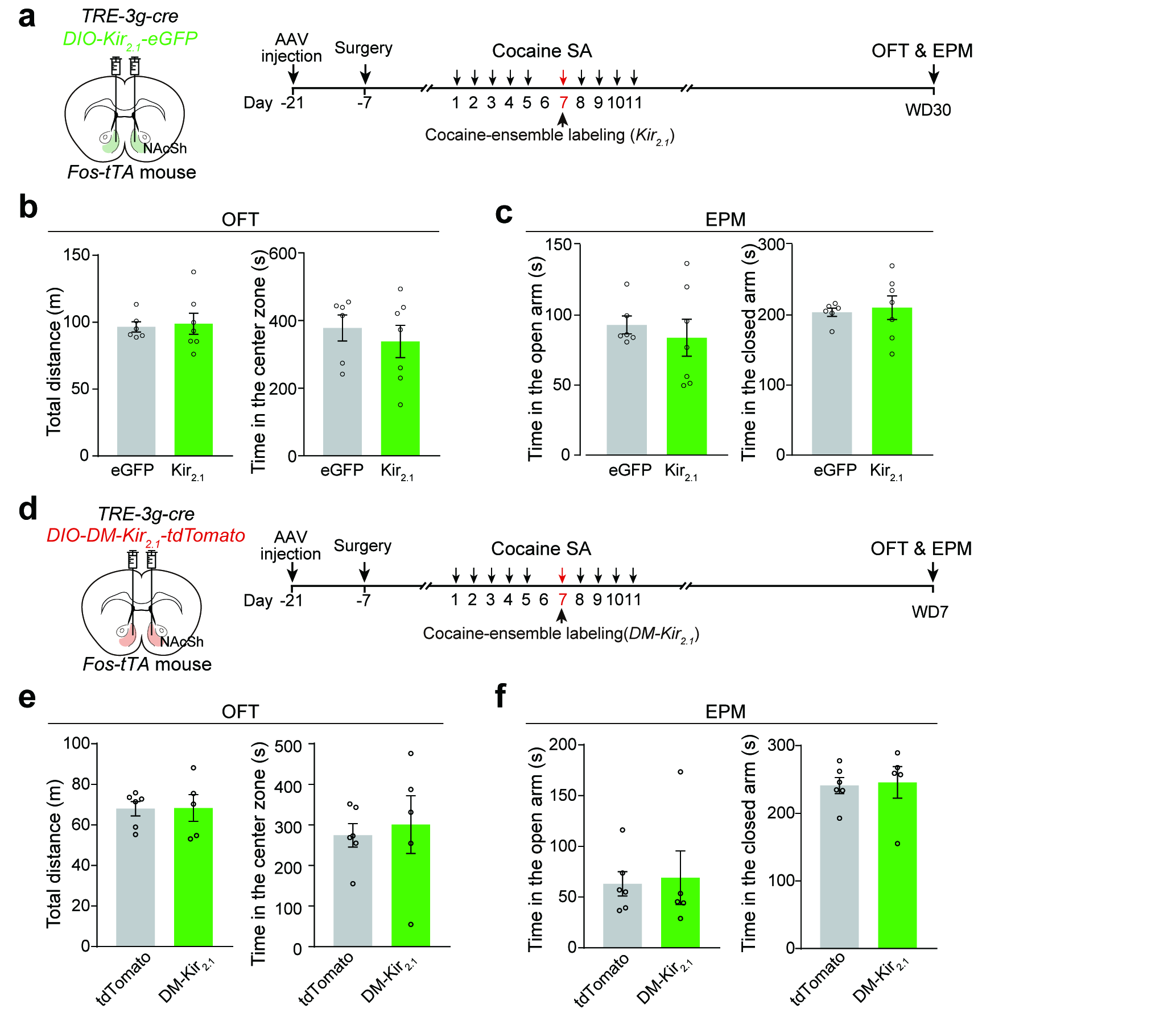

Supplement: Supplementary file 10 — Supplementary Figure 9 [file 41380_2022_1884_MOESM10_ESM.tif]
